# The Cervicovaginal Microbiota-Host Interaction Modulates Chlamydia trachomatis Infection

**DOI:** 10.1128/mBio.01548-19

**Published:** 2019-08-06

**Authors:** Vonetta L. Edwards, Steven B. Smith, Elias J. McComb, Jeanne Tamarelle, Bing Ma, Michael S. Humphrys, Pawel Gajer, Kathleen Gwilliam, Alison M. Schaefer, Samuel K. Lai, Mishka Terplan, Katrina S. Mark, Rebecca M. Brotman, Larry J. Forney, Patrik M. Bavoil, Jacques Ravel

**Affiliations:** a Institute for Genome Sciences, University of Maryland School of Medicine, Baltimore, Maryland, USA; b Department of Microbiology and Immunology, University of Maryland School of Medicine, Baltimore, Maryland, USA; c Biostatistics, Biomathematics, Pharmacoepidemiology and Infectious Diseases, Institut Pasteur, INSERM, Université de Versailles-Saint-Quentin-en-Yvelines, Versailles, France; d Department of Microbiology and Immunology, University of North Carolina at Chapel Hill, Chapel Hill, North Carolina, USA; e Department of Obstetrics and Gynecology, University of Maryland School of Medicine, Baltimore, Maryland, USA; f Department of Epidemiology and Public Health, University of Maryland School of Medicine, Baltimore, Maryland, USA; g Department of Biological Sciences, University of Idaho, Moscow, Idaho, USA; h Department of Microbial Pathogenesis, University of Maryland School of Dentistry, Baltimore, Maryland, USA; Icahn School of Medicine at Mount Sinai; University of Virginia; University of California, Irvine

**Keywords:** *Lactobacillus*, epigenetic, lactic acid, microbiome, proliferation, sexually transmitted infection

## Abstract

The mechanism(s) by which *Lactobacillus*-dominated cervicovaginal microbiota provide a barrier to Chlamydia trachomatis infection remain(s) unknown. Here we evaluate the impact of different *Lactobacillus* spp. identified via culture-independent metataxonomic analysis of C. trachomatis-infected women on C. trachomatis infection in a three-dimensional (3D) cervical epithelium model. *Lactobacillus* spp. that specifically produce d(−) lactic acid were associated with long-term protection against C. trachomatis infection, consistent with reduced protection associated with Lactobacillus iners, which does not produce this isoform, and with decreased epithelial cell proliferation, consistent with the observed prolonged protective effect. Transcriptomic analysis revealed that epigenetic modifications involving histone deacetylase-controlled pathways are integral to the cross talk between host and microbiota. These results highlight a fundamental mechanism whereby the cervicovaginal microbiota modulates host functions to protect against C. trachomatis infection.

## Abstract (precis)

The vaginal microbiota is believed to protect women against Chlamydia trachomatis, the etiologic agent of the most prevalent sexually transmitted infection (STI) in developed countries. The mechanism underlying this protection has remained elusive. Here, we reveal the comprehensive strategy by which the cervicovaginal microbiota modulates host functions to protect against chlamydial infection, thereby providing a novel conceptual mechanistic understanding. Major implications of this work are that (i) the impact of the vaginal microbiota on the epithelium should be considered in future studies of chlamydial infection and other STIs and (ii) a fundamental understanding of the cervicovaginal microbiota’s role in protection against STIs may enable the development of novel microbiome-based therapeutic strategies to protect women from infection and improve vaginal and cervical health.

## INTRODUCTION

The human vaginal microbiota participates in a mutualistic relationship with the host and play a role in modulating the risk to acquiring and transmitting sexually transmitted infections (STIs) (1–3). These include Chlamydia trachomatis infections, the most widespread STIs in the United States and worldwide (4), which, if left untreated, can cause serious sequelae such as pelvic inflammatory disease (PID) (5) and tubal factor infertility and ectopic pregnancy (6). In population-level metataxonomic surveys, at least five major types of vaginal microbiota or community state types (CSTs) have been identified (7). Four CSTs are dominated by one of four species of *Lactobacillus* (CST I, L. crispatus; CST II, L. gasseri; CST III, L. iners; CST V, L. jensenii), while CST IV is characterized by a paucity of *Lactobacillus* and the presence of a diverse array of strict and facultative anaerobes such as Gardnerella vaginalis, Atopobium vaginae, and *Prevotella* among others (7). CSTs dominated by *Lactobacillus* species are characterized by a low vaginal pH (<4.5), which is driven by lactic acid produced by the *Lactobacillus* spp. present (8, 9). Epidemiological studies relying on morphological evaluation of vaginal microbiota composition have shown that the presence of large numbers of *Lactobacillus* is associated with a lower risk of infection by sexually transmitted pathogens, including Neisseria gonorrhoeae, C. trachomatis, and HIV (10–13). Accordingly, lactic acid and low pH are believed to be the main factors underlying the properties that are protective against STIs.

The molecular mechanisms by which vaginal microbiota affect the risk of STI acquisition remain unknown. Recent studies have demonstrated that lactic acid can inhibit C. trachomatis infection of cultured cells (14). In addition, studies using C. trachomatis-infected cultured epithelial cells showed that culture supernatants of *Lactobacillus* spp. inhibit C. trachomatis infection and intracellular development (15). Interestingly, both the d(−) and l(+) isomers of lactic acid are found in the human vagina (16, 17), and the ratio of d(−) to l(+) lactic acid depends on the species of *Lactobacillus* that dominates the vaginal microbiota (16). The possibility that these isomers play differential roles in preventing C. trachomatis infection has not been explored. Moreover, there is no evidence for the direct inhibition of C. trachomatis by the vaginal microbiota; thus, one cannot exclude the possibility that vaginal microbiota impart resistance to infection by modulating host cellular functions. Host responses to vaginal microbiota in the context of chlamydial infection are poorly understood (18). One such mechanism may involve microbial modulation of human microRNAs (miRNAs). miRNAs are ∼20-mer oligonucleotides that have been shown to regulate a myriad of functions via translational inhibition (19). Their role in regulating host cell responses to vaginal microbiota has not been examined and may constitute an underappreciated host response mechanism.

We conducted a comprehensive culture-independent metataxonomic analysis on a cohort of women with genital C. trachomatis infection, pre- and postantibiotic treatment. This analysis informed experiments to examine the impact of *Lactobacillus* culture supernatants on the susceptibility of cultured cervical epithelial cells to C. trachomatis infection. An independent machine learning approach was used to identify miRNAs expressed *in vivo* in response to various vaginal CSTs, the miRNA targets, and potential functional outcomes on the host. This enabled us to highlight the mechanisms by which d(−) lactic acid produced by certain *Lactobacillus* spp. may act as a potent effector molecule causing decreased proliferation of epithelial cells and in turn protection against C. trachomatis infection. Deciphering the complexity of host-microbiota interactions to the cellular and molecular levels will contribute to a better understanding of the mechanisms by which different types of vaginal microbiota modulate risk to C. trachomatis infection. In time, this may enable the development of novel clinical strategies to reduce susceptibility to the pathogen.

## RESULTS

### Vaginal microbiota dominated by *L. iners* is associated with increased susceptibility to C. trachomatis infection.

**FIG 1:**
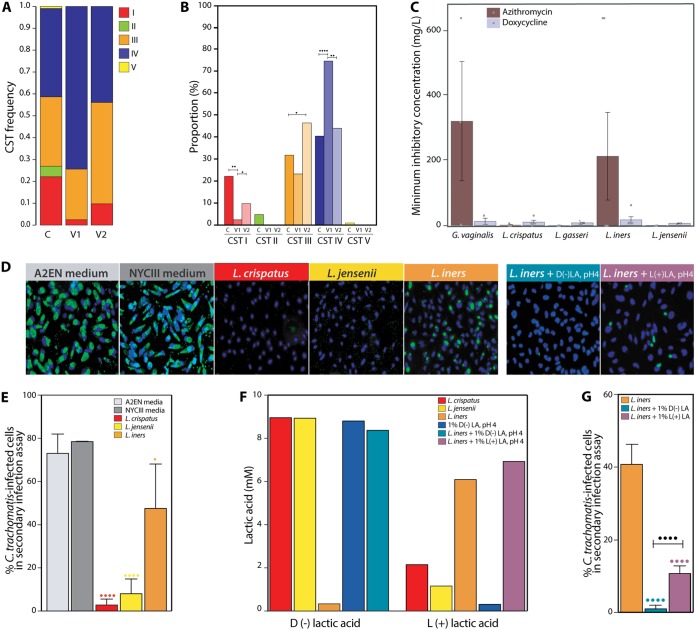
Effect of *Lactobacillus* spp. on C. trachomatis infectivity. (A) Frequency of vaginal CSTs in controls, at visit 1 (C. trachomatis positive) and visit 2 (C. trachomatis cleared). (B) Comparison of the proportions of each CST between control, visit 1, and visit 2. (C) Azithromycin and doxycycline MICs determined for strains of *G. vaginalis*, L. crispatus, L. gasseri, *L. iners*, and L. jensenii. (D) Secondary infection assay performed after A2EN 3D epithelial cells were preexposed prior to C. trachomatis infection to either cell culture medium (control), NYC III bacterial culture medium (control), or sterile-filtered culture supernatants from L. crispatus, L. jensenii, *L. iners* alone, and *L. iners* supplemented with 1% d(−) or l(+) lactic acid at pH 4 and visualized by fluorescence microscopy. Epithelial cell nuclei are stained blue while C. trachomatis inclusions are stained green. Representative images are shown. (E) Percentage of C. trachomatis-infected epithelial cells in secondary infection assays performed after A2EN 3D epithelial cell models were preexposed to control medium or culture supernatants from L. crispatus, L. jensenii, and *L. iners* prior to C. trachomatis infection. Results are from three independent experiments. (F) d(−) and l(+) lactic acid concentrations in the culture supernatants obtained from *Lactobacillus* spp. and chemical solutions of 1% d(−) and l(+) lactic acid. (G) Percentage of C. trachomatis-infected epithelial cells in secondary infection assays from A2EN 3D epithelial cell models preexposed prior to C. trachomatis infection to *L. iners* culture supernatant alone and *L. iners* culture supernatant supplemented with 1% d(−) or l(+) lactic acid at pH 4. Results are from three independent experiments. Statistical significance is shown as follows: one dot, *P* value < 0.05; two dots, *P* value < 0.01; three dots, *P* value < 0.001; and four dots, *P* value < 0.0001. Colored dots indicate pairwise comparison to A2EN medium, while black dots represent statistical significances obtained from ANOVAs comparing all four treatments.

The composition and structure of vaginal microbiota of 150 women were determined at the time of C. trachomatis diagnosis (visit 1) and 3 months after successful azithromycin treatment (visit 2). Analysis showed that CST IV and CST III were the most prevalent types at visit 2 (Fig. 1A). In response to treatment, the proportion of CST IV vaginal microbiota decreased from 74.4% to 43.9% (*P = *0.0029), while that of CST III increased from 23.2% to 46.3% (*P = *0.0048). Analysis of CST frequency in vaginal microbiota posttreatment revealed a statistically significant overrepresentation of CST III compared to a control group of 99 asymptomatic, apparently healthy women recruited at the same clinical site (7) (*P = *0.047) (Fig. 1A and B). These findings led us to postulate that (i) antibiotic treatment impacts the composition of the vaginal microbiota by favoring bacteria resistant to azithromycin and (ii) microbiota dominated by *L. iners* (CST III) provide suboptimal protection against C. trachomatis infection.

**FIG 2:**
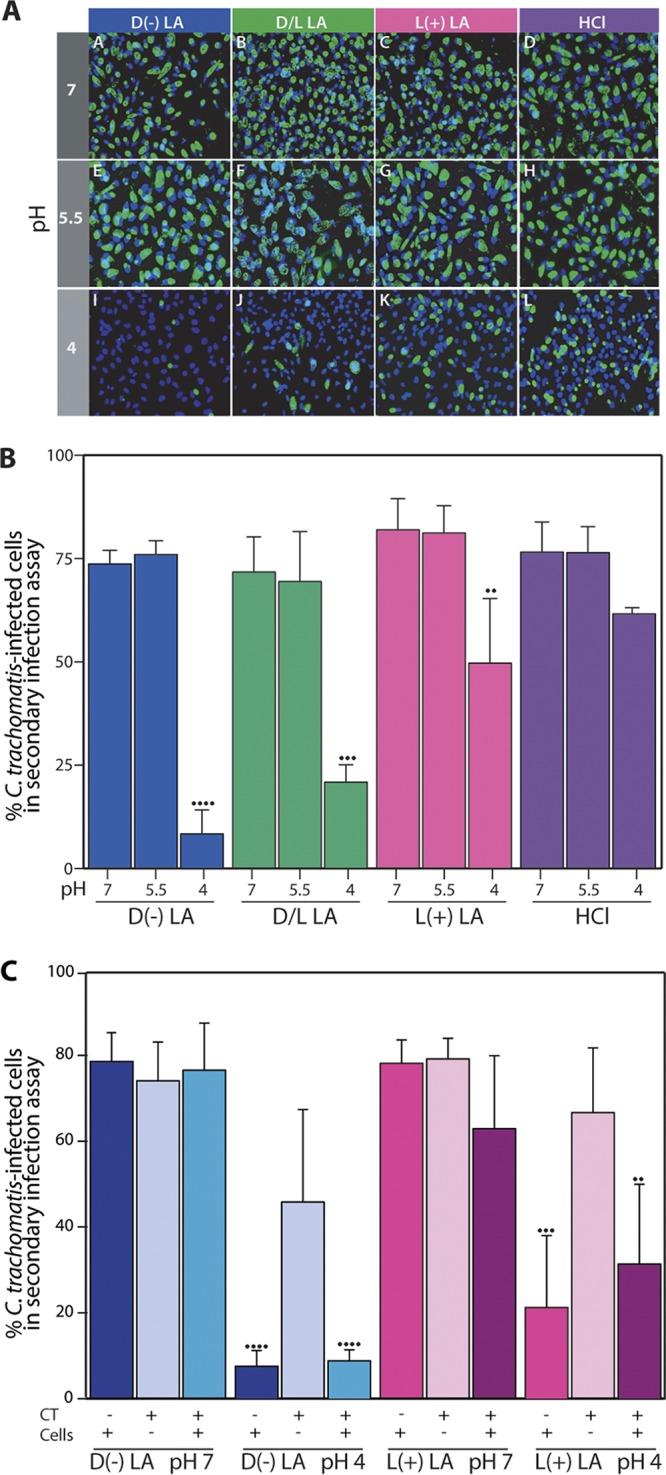
Effect of d(−), d/l racemic mixture, and l(+) lactic acid on C. trachomatis infectivity. (A) Secondary infection assay of A2EN 3D epithelial cells preexposed prior to C. trachomatis infection to lactic acid or HCl at various concentrations and hence pHs. Representative images are shown. (B) Percentages of C. trachomatis-infected epithelial cells in secondary infection assays are shown; results are from three independent experiments. (C) Percentage of C. trachomatis-infected epithelial cells in secondary infection assays after A2EN 3D epithelial cell models were either preexposed to d(−) or l(+) lactic acid at pH 4 or 7 and infected with C. trachomatis or not exposed but infected with C. trachomatis preexposed to d(−) or l(+) lactic acid at pH 4 or 7 or both C. trachomatis and cells were preexposed to d(−) or l(+) lactic acid at pH 4 or 7. Results are from three independent experiments. Statistical significance is shown as follows: one dot, *P* value < 0.05; two dots, *P* value < 0.01; three dots, *P* value < 0.001; and four dots, *P* value < 0.0001.

We tested the growth-inhibitory potential of azithromycin and doxycycline, respectively, the first- and second-line antibiotics for the treatment of C. trachomatis infection, toward key vaginal bacteria *in vitro*. Low concentrations (0.06 to 8 mg liter^−1^, average 1.0 mg liter^−1^) of azithromycin inhibited the growth of all L. crispatus, L. jensenii, and L. gasseri strains tested but had minimal effect on most strains of *L. iners* and *G. vaginalis* at the maximum concentration of antibiotic tested (640 mg liter^−1^) (Fig. 1C). Interestingly, doxycycline was equally inhibitory to all strains of all vaginal bacterial species tested (2 to 32 mg liter^−1^, average 8.7 mg liter^−1^) (Fig. 1C). Thus, azithromycin treatment contributes to shaping the vaginal microbiota, leading to microbiota dominated by *L. iners* or *G. vaginalis*. The latter is significant because the occurrence of microbiota depleted of *Lactobacillus* and comprising strict and facultative anaerobes such as *G. vaginalis* is a known risk factor for C. trachomatis infection (3). In addition, epidemiological studies have recently shown that *L. iners* appears to be associated with an increased risk of C. trachomatis infection (20). We experimentally confirmed this finding by measuring the abundance of infectious C. trachomatis particles produced by C. trachomatis-infected mucus-producing A2EN cervical epithelial cells (21) that had been preexposed to culture supernatants of various *Lactobacillus* spp. for 30 min. L. crispatus and L. jensenii supernatants conferred protection against C. trachomatis infection (24- and 9-fold decrease, respectively) relative to untreated A2EN cells or cells preexposed to bacterial culture (NYC III) medium. In contrast, *L. iners* supernatants caused only a 1.5-fold reduction in infection (Fig. 1D and E). A similar result was obtained when total number of inclusions were counted in this secondary infection model system (see Fig. S1A in the supplemental material). Further, to validate the secondary infection assay as an appropriate/effective surrogate for evaluating the level of C. trachomatis infection obtained on the three-dimensional (3D) A2EN epithelial cell model, we compared the trends observed in both the primary 3D infection and secondary infection. We found that proportionally similar trends were obtained when the total number of inclusions on the 3D A2EN primary infection model was compared to the percent chlamydia-infected cells in the secondary infection (Fig. 2 and Fig. S1B and C). We thus have used the secondary infection assay as our readout for this study.

10.1128/mBio.01548-19.2 FIG S1 (A) Effect of *Lactobacillus* culture supernatants on the total number of inclusions. (B and C) Direct effect of lactic acid on C. trachomatis infection in the 3D A2EN epithelial cell model. (D and E) Effect of pH driven by increasing the concentration of d(−) lactic acid or by maintaining the concentration of d(−) lactic acid and adjusting pH with NaOH. (F) Effect of d(−), d/l racemic mixture, or l(+) lactic acid on C. trachomatis serovar D infectivity. (G to I) A2EN epithelial cell viability after incubation with lactic acid at pH 4 or pH 7 or *Lactobacillus* culture supernatants. Download FIG S1, PDF file, 0.8 MB. Copyright © 2019 Edwards et al. 2019 Edwards et al. This content is distributed under the terms of the Creative Commons Attribution 4.0 International license.

Together, these findings indicate that azithromycin, while effective in the treatment of C. trachomatis infections, drives in part the formation of microbiota associated with increased risk of reinfection. However, these experiments cannot exclude that resolution of the C. trachomatis infection also contributes to the microbiota returning to a state corresponding to that found prior to infection, and combination of the two factors is likely. Nonetheless, the microbiota patterns observed may partly explain the high rates of reinfection previously reported (22–24). To investigate the molecular basis of the association between *L. iners* and increased risk of C. trachomatis infection, we evaluated the differential contribution of both lactic acid isomers to the protection against C. trachomatis infection.

### Supplementation of *L. iners* culture supernatants with d(−) lactic acid provides protective properties against C. trachomatis infection.

Previous studies have shown that L. crispatus, L. jensenii, and *L. iners* each produce different amounts of d(−) and l(+) lactic acid isomers (17). Consistent with these studies, culture supernatants of L. crispatus contained four times more d(−) than l(+) lactic acid and those of L. jensenii contained approximately nine times more d(−) than l(+) lactic acid, while *L. iners* culture supernatants contained almost exclusively l(+) lactic acid (Fig. 1F). The pH of all culture supernatants was about 4. We hypothesized that the different amounts of lactic acid isomers produced by each species of *Lactobacillus* might at least partially account for their protective properties against C. trachomatis infection. We tested the hypothesis that d(−) lactic acid specifically inhibits C. trachomatis infection by supplementing *L. iners* culture supernatants with 1% d(−) or l(+) lactic acid buffered to pH 4. As predicted, supplementation of *L. iners* culture supernatants with 1% d(−) lactic acid had inhibitory properties comparable to those of L. crispatus culture supernatants. Taken together, these results indicate that d(−) lactic acid produced by vaginal *Lactobacillus* spp. is a more potent inhibitor of C. trachomatis infection than l(+) lactic acid, the isomer associated with *L. iners*-dominated microbiota (Fig. 1E and G).

### Isomers of lactic acid significantly affect C. trachomatis infection in a pH-dependent manner.

In subsequent experiments, we showed that isomers of lactic acid modulate C. trachomatis infection in a pH-dependent manner. A2EN cervical epithelial cells were preexposed to d(−), l(+), and racemic lactic acid buffered to pH 7.0, 5.5, or 4.0. Inhibition of C. trachomatis infection by lactic acid was greatest at pH 4, while pH alone (HCl at pH 4) did not significantly affect infectivity (Fig. 2A and B), indicating that the protonated form of lactic acid is the active molecule. Similar results were achieved when the experimental pH was reduced from 7 to 4 by increasing the concentration of d(−) lactic acid from 15 mM to 28 mM (Fig. S1D and E). In these experiments, neither d(−), l(+), nor racemic lactic acid; HCl; or culture supernatants affected epithelial cell viability (at least 94% cell viability was observed) (Fig. S1G to I). These results demonstrate that d(−) lactic acid at pH 4 has the highest capacity to reduce C. trachomatis infection in a 3D model of A2EN cervical epithelial cells.

### Preexposure to lactic acid lowers host cell susceptibility to infection and does not affect the barrier properties of cervical mucus.

We then tested whether lactic acid exerts its effect(s) on host cells or directly on C. trachomatis infectious particles. C. trachomatis infection levels were compared in experiments where A2EN epithelial cells and/or C. trachomatis had been independently preexposed to d(−) or l(+) lactic acid at pH 7 and pH 4. Preexposure of C. trachomatis to either isomer of lactic acid at pH 7 or pH 4 did not protect epithelial cells against C. trachomatis infection (Fig. 2C). However, infectivity was reduced if host cells were preexposed to d(−) or l(+) lactic acid at pH 4. Combined, these results suggest that lactic acid does not directly affect C. trachomatis infectious particles but potentially inhibits a host-pathogen interaction required for the initiation or progression of infection.

We next evaluated the possibility that lactic acid modulates the barrier properties of cervicovaginal mucus toward penetrating C. trachomatis infectious particles. Previous work showed that endogenous d(−) lactic acid levels in native cervicovaginal mucus correlated with HIV mobility, with a statistically significant correlation between lower d(−) lactic acid content and greater HIV mobility in native secretions (25). Cervicovaginal secretions from 34 reproductive-age women were used to measure the mobility of fluorescently labeled C. trachomatis particles relative to the concentration of each lactic acid isomer. Under both native acidic and neutralized conditions, the mobility of C. trachomatis particles was not appreciably correlated with the endogenous d(−) lactic acid content: C. trachomatis particles were effectively trapped at native pH, while substantially greater motion heterogeneity was observed at neutral pH (Fig. S2). Thus, variations in endogenous d(−) lactic acid do not appear to correlate with differences in the barrier properties of cervicovaginal mucus in a way that could modulate C. trachomatis infection.

10.1128/mBio.01548-19.3 FIG S2 Mobility of fluorescently labeled C. trachomatis serovar L2 in native and pH-neutralized cervicovaginal mucus (CVM) compared to d-lactic acid concentrations. Download FIG S2, PDF file, 0.1 MB. Copyright © 2019 Edwards et al. 2019 Edwards et al. This content is distributed under the terms of the Creative Commons Attribution 4.0 International license.

### The effect of preexposing host cells to lactic acid or *Lactobacillus* culture supernatants is long-lasting.

**FIG 3:**
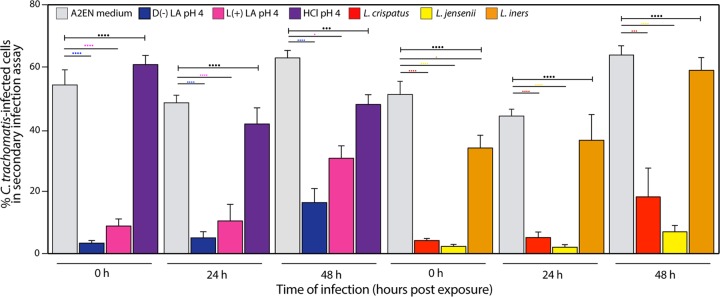
Long-term effect of lactic acid and *Lactobacillus* culture supernatants on C. trachomatis infectivity. Percentage of C. trachomatis-infected epithelial cells in secondary infection assays after A2EN 3D epithelial cell models were preexposed to lactic acid; HCl; or L. crispatus, L. jensenii, and *L. iners* culture supernatants prior to C. trachomatis infection performed at 0 h, 24 h, and 48 h. Results are from three independent experiments. Statistical significance is shown as follows: one dot, *P* value < 0.05; two dots, *P* value < 0.01; three dots, *P* value < 0.001; and four dots, *P* value < 0.0001. Colored dots correspond to pairwise comparison to A2EN medium, and black dots represent statistical significances obtained from ANOVAs comparing the four treatments.

To assess the long-term effect of lactic acid, we evaluated C. trachomatis infectivity 24 h and 48 h after exposure to d(−) lactic acid, l(+) lactic acid (pH 4), or *Lactobacillus* culture supernatants. Surprisingly, for up to 48 h, C. trachomatis infectivity toward cells preexposed to lactic acid or *Lactobacillus* culture supernatants was significantly reduced (Fig. 3). Exposure of cells to L. crispatus and L. jensenii culture supernatants reduced C. trachomatis infection 24 h postexposure (8- to 21-fold). This was similar to the reduction observed when cells were infected immediately after exposure to culture supernatants (12- to 22-fold). In contrast, exposure to *L. iners* culture supernatants did not produce any significant reduction in infectivity at any time postexposure compared to medium only (Fig. 3). These results show that A2EN cells exposed to lactic acid or L. crispatus and L. jensenii culture supernatants maintain long-term protection against C. trachomatis infection.

### *Lactobacillus*-dominated vaginal microbiota affect epithelial cell cycling *in vivo*.

**FIG 4:**
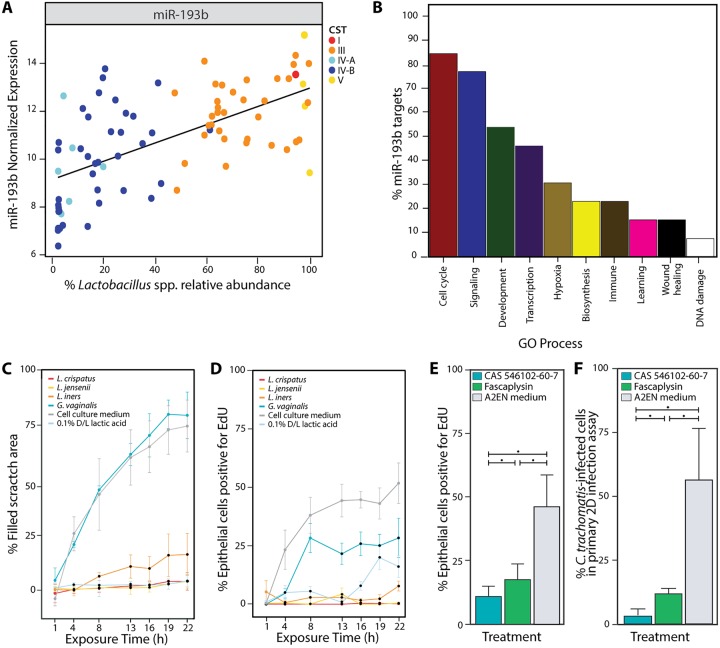
The composition of the vaginal microbiota affects cell cycling functions and C. trachomatis infection. (A) Normalized miR-193b expression in relation to *in vivo Lactobacillus* relative abundance. Each point is colored according to the CST of the samples from which it is derived. (B) Percentage of miR-193b gene targets mapped to Gene Ontology Direct major processes. Evaluation of the effect of culture supernatant on migration as evaluated by scratch assay of VK2 epithelial cells. (C) Percent filled scratch area (compared to 0 h) after exposure to L. crispatus, L. jensenii, *L. iners*, and *G. vaginalis* culture supernatants or lactic acid for 22 h. (D) Evaluation of the effect of culture supernatant on proliferation evaluated by EdU assay. Percentage of VK2 epithelial cells positive for EdU nucleobases at each time point across the 22-h assay. (E) Percentage of A2EN epithelial cells positive for EdU nucleobases after exposure to CDK4 inhibitors CAS 546102-60-7 and Fascaplysin or cell culture medium and evaluation by EdU assay. (F) Percentage of C. trachomatis-infected cells in the primary 2D A2EN epithelial cells preexposed to CDK4 inhibitors or cell culture medium performed on coverslips. Statistical significance is shown as follows: single dot, *P* value < 0.05. For panels C and D, statistically significant time points (*P* value < 0.05) are represented with black points.

The prolonged protective effects of lactic acid and *Lactobacillus* culture supernatants on C. trachomatis infection suggest mechanisms involving changes in the expression of host cellular pathways. Thus, we sought to characterize host responses to different types of vaginal microbiota and their relevance to C. trachomatis infection. We explored the effect of vaginal microbiota composition on epithelial cell function *in vivo* by characterizing the types and abundance of human microRNAs (miRNAs), which have been shown to regulate a myriad of functions via translational inhibition (19). We successfully performed small RNA transcriptome sequencing (small RNA-seq) on 83 samples collected from 16 women over a 10-week period (Table S1A). These represented vaginal microbiota that were either dominated by *Lactobacillus* spp. or lacked *Lactobacillus* spp. and instead were comprised of a wide array of strict and facultative anaerobes (Text S1 and Table S1B). We found there was no biased correlation between read numbers and sequencing runs or participants (Fig. S3). A Random Forest model using 169 nonzero log_2_-transformed miRNA read counts as inputs (Table S1C) showed that miR-193b, miR-203b, miR-320b-1, miR-223, and miR-183 were best able to predict the relative abundance of *Lactobacillus* spp. (Fig. 4A; see also Fig. S4A and Table S1D). miR-193b, miR-183, and miR-223 are involved in transcription, cell cycle, signaling, development, and hypoxia, while miR-203b and miR-320b-1 do not have experimentally validated targets (Fig. S4B and Table S1E). The expression of miR-193b was among the highest (log_2_-normalized reads >5 in all samples) and most positively correlated with *Lactobacillus* relative abundance (Fig. 4A). miR-193b function is broadly annotated as a tumor suppressor since it has been shown to inhibit the expression of several genes associated with cell proliferation and metastasis. Multiple targets have been identified in a variety of cell lines (Table S1E) (19, 26–34).

10.1128/mBio.01548-19.1 TEXT S1 Supplemental materials and methods and references. Download Text S1, PDF file, 0.2 MB. Copyright © 2019 Edwards et al. 2019 Edwards et al. This content is distributed under the terms of the Creative Commons Attribution 4.0 International license.

10.1128/mBio.01548-19.4 FIG S3 Quality control (QC) of small RNA-seq samples. Download FIG S3, PDF file, 0.3 MB. Copyright © 2019 Edwards et al. 2019 Edwards et al. This content is distributed under the terms of the Creative Commons Attribution 4.0 International license.

10.1128/mBio.01548-19.5 FIG S4 (A and B) Association between *Lactobacillus* relative abundance, miRNAs, and predicted function. (C to F) miR-193b expression and CCND1 levels in VK2 epithelial cells. (G and H) VK2 cell migration and proliferation assay. Download FIG S4, PDF file, 0.9 MB. Copyright © 2019 Edwards et al. 2019 Edwards et al. This content is distributed under the terms of the Creative Commons Attribution 4.0 International license.

10.1128/mBio.01548-19.7 TABLE S1 (A) Small RNA-seq miRNA raw read counts for each sequenced sample. (B) Small RNA-seq library preparation, sequencing and alignment statistics, QC analysis annotation, and subject metadata used in analysis. (C) Post-QC log_2_-normalized small RNA-seq counts used to test and train Random Forest model. (D) Importance metrics and *P* values for the Random Forest variable selection. (E) Experimentally validated gene targets for each of the miRNAs for which expression is associated with *Lactobacillus* proportion. (F) Ingenuity Pathway Analysis (IPA) results (Z-scores). Download Table S1, XLSX file, 1.0 MB. Copyright © 2019 Edwards et al. 2019 Edwards et al. This content is distributed under the terms of the Creative Commons Attribution 4.0 International license.

The well-characterized functions of miR-193b coupled with its relationship to the vaginal microbiota composition strongly suggested that miR-193b inhibits vaginal epithelial cell migration and proliferation when exposed to *Lactobacillus* culture supernatants. One experimentally validated target of miR-193b is the cell cycle checkpoint regulator cell cyclin D1 (CCND1), which is involved in promoting progression through G_1_-S phase, thus facilitating proliferation (35, 36). To examine whether miR-193b levels correlate with altered CCND1 expression, we used quantitative PCR (qPCR) and immunoblotting to measure the levels of miR-193b and CCND1 in cultured VK2 vaginal epithelial cells that had been exposed to 20% (vol/vol) culture supernatants from L. crispatus, L. jensenii, *L. iners*, and *G. vaginalis* as a non-*Lactobacillus* control or racemic lactic acid over a 22-h period. The relative expression of miRNA-193b after exposure to culture supernatants or racemic lactic acid reflected our *in vivo* findings wherein miR-193b expression was positively associated with vaginal microbiota dominated by a *Lactobacillus* species (Fig. S4C). Consistent with this finding, higher levels of miR-193b were associated with lower levels of CCND1 (Fig. S4C and D). Normalized CCND1 production at 13 h was 85%, 75%, and 57% lower than control when VK2 cells were exposed to L. crispatus or L. jensenii culture supernatants or racemic lactic acid, respectively. In contrast, CCND1 production was 34% and 8% higher in cells preexposed to *L. iners* and *G. vaginalis* culture supernatants, respectively, than to medium-only controls. Similar trends were observed after 22 h of exposure to culture supernatants (Fig. S4E). Finally, CCND1 production in VK2 cells transfected with an miR-193b mimic was reduced to 52% of that in cells transfected with a scrambled sequence, supporting previous evidence that miR-193b specifically targets CCND1 (Fig. S4F) (28, 33).

### *Lactobacillus* culture supernatants modulate vaginal epithelial cell migration and proliferation.

To determine if predicted miRNA functions translated into phenotypic differences in cultured VK2 vaginal epithelial cells, we tested the ability of various culture supernatants or racemic lactic acid to inhibit cell migration or proliferation over a 22-h time course. Relative to cell culture medium, VK2 cells exposed to culture supernatants of L. crispatus, L. jensenii, or *L. iners* or racemic lactic acid showed significantly less cell migration (Fig. 4C and Fig. S4G) and cell proliferation (Fig. 4D and Fig. S4H). The decrease in DNA synthesis (i.e., cell proliferation) in VK2 cells exposed to *G. vaginalis* culture supernatants was less and not as significant as that observed with *Lactobacillus* spp. or racemic lactic acid (Fig. 4D and Fig. S4H). The observed differences in cell migration and proliferation were not explained by differences between culture media for *Lactobacillus* spp. (NYC III) or for *G. vaginalis* (tryptic soy broth [TSB]) (data not shown). *L. iners* culture supernatants were less able to induce miRNA-193b expression or inhibit cell migration and proliferation relative to L. crispatus or L. jensenii culture supernatants (Fig. 4A, C, and D and Fig. S4C, G, and H). Taken together, the evidence suggests that vaginal microbiota dominated by a *Lactobacillus* spp. producing d(−) lactic acid are associated with elevated miR-193b expression in vaginal epithelial cells and, consequently, with decreased vaginal epithelial cell migration and proliferation. These results suggest that CCND1 production was decreased after exposure to L. crispatus and L. jensenii culture supernatants through increased miR-193b expression and targeting of CCND1. The *L. iners* discordant relationship between increased CCND1 protein expression and the absence of proliferation may be partially explained by the inability of *L. iners* to induce a globally sustained effect on proliferation, unlike L. crispatus and L. jensenii.

### Epithelial cell proliferation is required for efficient C. trachomatis infection.

The findings described above led to the hypothesis that epithelial cell proliferation may play a role in susceptibility to C. trachomatis infection. To test this, we used the validated inhibitors of CDK4/cyclin D1, CAS 546102-60-7 (37) and fascaplysin (38), to block A2EN cell proliferation before challenge with C. trachomatis. Compared to culture medium alone, proliferation decreased 35.2% (*P = *6.1 × 10^−5^) and 28.5% (*P = *3.5 × 10^−4^) when cells were exposed to either CAS 546102-60-7 or fascaplysin, respectively (Fig. 4E and Fig. S4C). Consistent with these findings, C. trachomatis infection of A2EN cells decreased 53.4% (*P = *3.3 × 10^−4^) and 44.6% (*P = *1.0 × 10^−3^), respectively (Fig. 4F). Cell viability was not affected by exposure to the inhibitors (Fig. S4C). These results suggest that epithelial cell proliferation is required for efficient C. trachomatis infection.

### *Lactobacillus* spp. control a global transcriptional network and provide protection from C. trachomatis infection.

Given that the cell cycle is regulated by multiple mechanisms, transcriptome sequencing (RNA-seq) was used to identify differentially expressed cell cycle genes after exposure to *Lactobacillus* and *G. vaginalis* culture supernatants for 4 h, 13 h, or 22 h. Genes that were differentially expressed at each time point postexposure were identified by comparing cells exposed to either culture supernatants or cell culture medium. Among the 28 most commonly activated or repressed canonical host pathways, 11 were related to the cell cycle. L. crispatus and L. jensenii culture supernatants generally repressed cell proliferation pathways (Table S1F), whereas *L. iners* or *G. vaginalis* supernatants did not.

**FIG 5:**
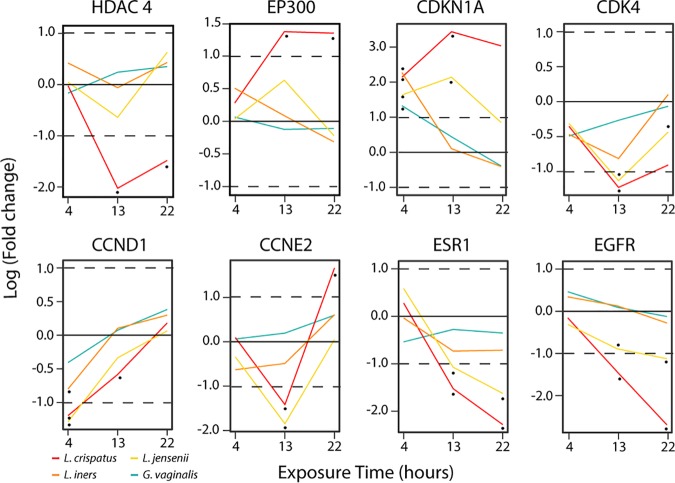
Longitudinal relative expression patterns for selected cell cycle and chromatin remodeling genes. Shown are gene log_2_ fold change values for 4 h, 13 h, and 22 h in VK2 cells exposed to L. crispatus, L. jensenii, *L. iners*, or *G. vaginalis* culture supernatants or cell culture medium. Horizontal lines indicate log_2_ fold changes of −1, 0, and 1. Statistical significance is shown as follows: single dot, FDR < 0.01.

The observation that multiple cell proliferation pathways were repressed upon exposure to L. crispatus and L. jensenii culture supernatants suggests that the cell cycle might be regulated by a global mechanism. One such mechanism could involve histone deacetylase enzymes (HDACs), which regulate gene transcription by chromatin remodeling (39). When the temporal expression of HDAC4 was analyzed, we observed a decreased log fold change (logFC) pattern 13 h after exposure to L. crispatus or L. jensenii culture supernatants (Fig. 5). Additionally, the opposite impact was observed for the longitudinal expression pattern of histone acetyltransferase enzyme (HAT) E1A binding protein p300 (Fig. 5). HDAC4 negatively regulates the transcription of cyclin-dependent kinase inhibitor 1A (CDKN1A) (40), which then inhibits the activity of cyclin-dependent kinase 4 (CDK4) and thus the cell cycle. As expected, in VK2 cells that were exposed to L. crispatus and L. jensenii culture supernatants for 13 h (Fig. 5) the logFC expression of CDKN1A was highest, while the logFC expression of CDK4 was lowest. Although CCND1 gene logFC increased over time in all cases, it increased significantly less for cells exposed to L. crispatus and L. jensenii culture supernatants (Fig. 5). Cell cyclin E2 (CCNE2), a regulator of late G_1_/S-phase cell progression, shows decreased expression at 13 h versus cell culture medium upon exposure to L. crispatus and L. jensenii culture supernatants relative to *L. iners* or *G. vaginalis* culture supernatants (Fig. 5). The observed 13-h logFC expression profiles of CDKN1A, CDK4, CCND1, and CCNE2 and the differential transcription of histone modification genes HDAC4 and EP300 suggest a global regulation of CDKN1A and the cell cycle after exposure to L. crispatus or L. jensenii culture supernatants. Estrogen receptor alpha (ESR1) is another target of miR-193b, which, when expressed, induces cell growth in the presence of estrogen in mouse and human vaginal cells (41–43). Figure 5 shows reduced differential expression (false-discovery rate [FDR] of <0.01) of ESR1 in cells exposed to L. crispatus and L. jensenii culture supernatants. Previous studies have shown that epidermal growth factor receptor 1 (EGFR) is required for internalization of chlamydial elementary bodies (EBs) into host cells (44). EGFR’s logFC expression decreased from 4 h to 22 h (Fig. 5) in cells exposed to L. crispatus and L. jensenii culture supernatants with an FDR of <0.01 at 13 h and 22 h postexposure. This suggests that decreased EGFR expression may be mediated by components of *Lactobacillus* culture supernatants, thereby providing an additional protective mechanism against C. trachomatis internalization and infection.

## DISCUSSION

Epidemiological studies have demonstrated a strong, statistically significant association between vaginal microbiota dominated by *Lactobacillus* spp. and decreased risk for STIs, including C. trachomatis infection (3). Conversely, a vaginal microbiota depleted of *Lactobacillus* spp. and consisting of various strict and facultative anaerobic bacteria is a significant risk factor for the acquisition of STIs. Recent *in vitro* and *in vivo* studies have shown that among vaginal *Lactobacillus* spp., *L. iners* is associated with lower protection against C. trachomatis infection than L. crispatus, L. gasseri, and L. jensenii (45, 46). Similar findings were reported in a cohort of Dutch women (20). The differences between *L. iners* and the other *Lactobacillus* spp. have often been attributed to the inability of *L. iners* to produce hydrogen peroxide. However, the antimicrobial properties of H_2_O_2_ *in vivo* have been recently questioned, and a role for lactic acid and its associated low pH has been proposed (47). In cultured cells, lactic acid was shown to have antichlamydial properties, believed to directly affect the infectious chlamydiae (47). Our results demonstrate that lactic acid does indeed play a major role in the antichlamydial properties of the vaginal microbiota by modulating host epithelial functions. More importantly however, d(−) lactic acid provided higher protection than l(+) lactic acid, consistent with the lower antichlamydial properties exhibited by *L. iners*, which does not produce d(−) lactic acid. Combined with the higher level of resistance of *L. iners* to azithromycin, a front-line antibiotic to treat C. trachomatis infection, our results identify a potentially major public health issue, in that treatment, while critical for curing the infection, does not favor restoration of a protective vaginal microbiota. Establishing a protective vaginal environment posttreatment would potentially greatly reduce the incidence of C. trachomatis recurrent infections or reinfections (e.g., the incidence of C. trachomatis*-*positive participants 3 months posttreatment was about 20% in the CHARM study). These findings support the need to develop adjunct therapy, such as the use of live biotherapeutics that would foster a protective vaginal environment, thereby reducing the risk for reinfection.

A significant result of this study highlights the potential for a “protective” vaginal microbiota to reduce the risk of C. trachomatis infection through the production of d(−) lactic acid well before exposure to the pathogen. In these experiments, a relatively short exposure (30 min) to L. crispatus and L. jensenii culture supernatants reduced the probability of infection for up to 24 h postexposure. The composition of the vaginal microbiota may undergo rapid dynamic shifts, resulting in the loss of *Lactobacillus* dominance, which can be short-lived or last for long periods (48, 49). The duration of these transitional states and the frequency at which they appear are thought to correlate with the risk of STIs, including C. trachomatis infection. Our results imply that a short-term shift (<48 h) might not dramatically increase the risk of C. trachomatis infection. For instance, the ∼8-h pH increase associated with ejaculation (50) would not necessarily increase the risk of C. trachomatis infection if the vaginal microbiota was dominated by d(−) lactic acid-producing *Lactobacillus* spp. prior to exposure to semen. Interestingly, the effect of pretreatment with culture supernatant was greater than that of d(−) or l(+) lactic acid at pH 4 alone, indicating that potentially culture supernatants contain other, yet-unknown factors contributing to protection against C. trachomatis infection.

C. trachomatis is an intracellular pathogen that must be internalized by target cells. One can envision several mechanisms by which the vaginal microbiota might be an obstacle to internalization. First, C. trachomatis infectious particles, the elementary bodies (EBs) may be directly disabled upon exposure to microbial metabolites. Second, access and attachment of EBs to the host cells may be reduced by microbially directed modifications of the external structures of the cells. Last, the internalization process might be blocked or compromised upon downregulation of factors and pathways that are essential for the remodeling of the epithelial cell plasma membrane, which is itself critical for C. trachomatis internalization. In this study, we showed that the first two possibilities are unlikely. Direct exposure of C. trachomatis EBs to lactic acid did not affect their infectious potential. Further, cervicovaginal mucus associated with vaginal microbiota dominated by d(−) lactic acid-producing *Lactobacillus* spp., which has been shown to act as a barrier to HIV access to target cells (25), did not alter the penetration properties of C. trachomatis EBs. Indeed, motility of EBs through mucus was independent of the lactic acid isomer ratio. Thus, vaginal microbiota metabolites do not appear to modulate the physicochemical properties of mucus to form an effective barrier against C. trachomatis infection.

The data presented in this study support the hypothesis that inhibition of epithelial cell proliferation is a mechanism initiated in part by d(−) lactic acid-producing *Lactobacillus* spp. that ultimately reduces susceptibility to C. trachomatis infection. Interestingly, three studies have reported that gastrointestinal lactic acid-producing bacteria and vaginal bacteria affect epithelial cell proliferation. Wound healing was significantly reduced upon 24-h exposure of HeLa cells to culture supernatants of *G. vaginalis* compared to those of *L. iners* (51). Conversely, migration of MS374 cells was increased upon exposure to L. crispatus culture supernatants relative to control cell culture medium (52). In another study, lactate and acetate produced by Lactobacillus casei and Bifidobacterium breve inhibited gut epithelial cell proliferation (53). Moreover, this inhibition was associated with the downregulation of cyclin D1 and cyclin E1 production, as observed in the present study. These reports provide mechanistic support for our proposed interplay between the microbiota and epithelial cell homeostasis and identify short-chain fatty acids, such as lactate or acetate, as potential mediators. The results reported here are consistent with how *L. casei* and *B. breve* affect gastrointestinal cell proliferation but differ from the reported antimigration effect of *G. vaginalis* on HeLa cells (51) and promigration effect of L. crispatus on MS374 cells (52). The cancerous cervical origin of both HeLa and MS374 cell lines likely compromises these cells as models to study migration or proliferation. Indeed, these cell lines probably carry mutations in cell cycle checkpoint systems as demonstrated by Matsuki et al. with gut epithelial cell lines (53). The VK2 epithelial cell line used in our study was derived in a manner similar to the noncancerous transformed murine intestinal crypt cell line m-ICcl2 ultimately used by Matsuki et al. (53) and is therefore not expected to have large disruptions of cell cycle checkpoint systems (54, 55). More importantly, the antiproliferative phenotype and antichlamydial properties of *Lactobacillus* culture supernatants could be mechanistically replicated using validated proliferation inhibitors. Our findings are further supported by evidence that epithelial cell shedding positively correlated with *G. vaginalis* and sialidase (produced by *G. vaginalis*) in a murine model (56). *In vivo* vaginal epithelial cell exfoliation was also strongly associated with vaginal microbiota lacking *Lactobacillus* spp. as defined by a Nugent score of >3 (57). This suggests that increased proliferation might act as a host defense mechanism against *G. vaginalis*, a bacterial species known for its adherent and biofilm-forming properties, which is often associated with bacterial vaginosis (58, 59). Increased epithelial cell shedding may contribute to a host-driven defense mechanism whereby “unwanted” adherent bacterial cells are cleared from the epithelial surface. In turn, this may give *Lactobacillus* spp. the opportunity to adhere to and colonize the new epithelial surfaces, thus effectively displacing *G. vaginalis* from the vaginal epithelium (60–62), leading to the restoration of epithelial homeostasis.

Upon infection, C. trachomatis regulates cell proliferation by affecting cell transcription, DNA repair mechanisms, and cyclin E, as well as phosphatidylinositol 3-kinase (PI3K), MEK, and extracellular signal-regulated kinase (ERK) growth signaling pathways (44, 63–65). Our results suggest that by controlling the cell cycle, the vaginal microbiota can counter C. trachomatis-induced proliferation that is required by the infecting C. trachomatis EBs, thus contributing to the observed reduction in susceptibility to C. trachomatis infection. L. crispatus and L. jensenii culture supernatants modulated the expression of multiple genes related to cell proliferation, including a decrease in histone deacetylase HDAC4 and an increase in its counterpart, histone acetylase (HAT) EP300. Histone deacetylases (HDACs), which repress gene transcription via deacetylation and chromatin condensation (66), negatively regulate the transcription of the cyclin-dependent kinase inhibitor 1A (CDKN1A, also known as p21) (40), which in turn inhibits the activity of cyclin-dependent kinase 4 (CDK4) and thus the cell cycle (67, 68). Downregulation of HDAC4 by L. crispatus and L. jensenii culture supernatants led to an increase of CDKN1A and lower proliferation. Interestingly, work in the gut has shown that the activity of HDACs can be inhibited by short-chain fatty acids (69). This is in keeping with our observation that the inhibitory activity was maximal upon exposure to d(−) lactic acid or to the culture supernatants of d(−) lactic acid-producing *Lactobacillus* species. *L. iners*, which lacks the ability to produce d(−) lactic acid, was unable to downregulate HDAC4 and thus did not reduce cell proliferation sufficiently to confer high protection against C. trachomatis infection. Conversely, *G. vaginalis* promoted cell proliferation and increased susceptibility to C. trachomatis infection. These results are consistent with the observed lack of protection afforded by *L. iners* culture supernatants against C. trachomatis infection of cultured cells and for that associated with *L. iners*- and *G. vaginalis*-containing vaginal microbiota in C. trachomatis-infected women (3). Interestingly, the rate of transmission after contact with a C. trachomatis-infected partner has been estimated to be 25 to 40% (70–72), a frequency fully consistent with that of the occurrence of vaginal microbiota CSTs lacking *Lactobacillus* spp. in reproductive-age women (7).

HAT EP300 regulates estrogen receptor 1 (ESR1) by mediating ligand binding to the estrogen receptor (73). ESR1 is known to mediate vaginal cell proliferation in the presence of estrogen and signaling by unknown growth factors (42, 43). The results presented here suggest a relationship between EP300 expression and ESR1 transcripts. In this model, ESR1 transcription would be downregulated by exposure to L. crispatus or L. jensenii but not to *L. iners* or *G. vaginalis*, suggesting that ESR1 regulation by EP300 contributes to the control of cell homeostasis. The observed antiproliferative effect of *Lactobacillus* spp. may counterbalance the action of estrogen on cell proliferation. In this context, the presence of estrogen could facilitate rapid proliferative transition of the epithelium in response to depletion of *Lactobacillus* spp. in the vaginal microbiota.

Taken together, the findings reported here suggest that d(−) lactic acid-producing *Lactobacillus* spp. modulate vaginal epithelial cell homeostasis by regulating a global transcriptional and functional network that includes epigenetic mechanisms that regulate gene expression, ultimately leading to reduction in cell cycling and protection against C. trachomatis infection. The results highlight the need to understand the mechanistic and functional underpinnings of the host response to the cervicovaginal microbiota. Such understanding is a prerequisite to the development of strategies to modulate these natural protective barriers to STI.

## MATERIALS AND METHODS

### Ethical approval.

All clinical samples were collected under approved University of Maryland-Baltimore Institutional Review Board (IRB) protocols HP-00042350, HP-00076730, and HP-00040935 for CHARM, SENTINEL, and UMB-HMP studies, respectively. Written informed consent was obtained from each participant. Participant demographics are shown in Table S2 in the supplemental material.

10.1128/mBio.01548-19.8 TABLE S2 Clinical study demographics. Download Table S2, XLSX file, 0.01 MB. Copyright © 2019 Edwards et al. 2019 Edwards et al. This content is distributed under the terms of the Creative Commons Attribution 4.0 International license.

### Cell culture models and growth conditions.

All cell culture supplies were obtained from Invitrogen unless otherwise stated. A2EN human cervical epithelial cells (kindly provided by A. J. Quayle) (74) were cultured at 37°C in 5% CO_2_ in EpiLife complete medium. BJ human fibroblast cells (ATCC CRL-2522) and HeLa human cervical epithelial cells (ATCC CRL-2) were maintained at 37°C in 5% CO_2_ in Dulbecco’s modified Eagle’s medium (DMEM), supplemented with 10% fetal bovine serum (FBS) (Sigma). VK2 vaginal epithelial cells (ATCC CRL-2616) were cultured in keratinocyte serum-free complete medium (Gibco) at 37°C in 5% CO_2_. Cell lines were authenticated (Fig. S5).

10.1128/mBio.01548-19.6 FIG S5 Cell validation certificate. Download FIG S5, PDF file, 0.9 MB. Copyright © 2019 Edwards et al. 2019 Edwards et al. This content is distributed under the terms of the Creative Commons Attribution 4.0 International license.

To construct the three-dimensional (3D) A2EN epithelial cell model, 24-well transwell inserts (Corning 3472) were basally coated with rat tail collagen type I (Corning 35423) and allowed to solidify for 3 days at 4°C. BJ fibroblasts (3 × 10^4^ cells) were seeded on the collagen and grown for 2 days. A2EN cervical epithelial cells (1 × 10^5^) were apically seeded and allowed to grow for 7 days. Cells were fed basally every other day to create an air interface on the epithelial cell surface. Two-dimensional (2D) epithelial cell culture models were prepared by seeding 5 × 10^4^ A2EN or VK2 cells on coverslips and allowing them to grow for 2 days before being used in the assays.

### Bacterial strains, culture supernatants, and lactic acid concentrations.

Bacterial strains used were L. crispatus ATCC 33197, ATCC 33820, ATCC 55844, and PTA-10138; L. jensenii ATCC 25258; L. gasseri ATCC 33323; *L. iners* ATCC 55195; and *G. vaginalis* ATCC 14018 and ATCC 49145. Strain *L. iners* DSMZ 13335 was obtained from the Leibniz Institute DSMZ. Strain *L. iners* UPII-60-B was purchased from BEI Resources (HM-131). Strains L. crispatus EM14; *L jensenii* VM044, EM04, and J1-2; and L. gasseri EM06, G7.2, G120.1, and G124.3 were isolated from clinical samples in the laboratory. Strains of *Lactobacillus* spp. were cultured in NYCIII medium, and *G. vaginalis* strains were cultured in TSB medium at 37°C under anaerobic conditions using a Coy anaerobic chamber. The medium composition is in Text S1.

Bacterial culture supernatants were prepared by inoculation of 1 × 10^8^ cells of an overnight culture in 10 ml of fresh NYC III or TSB medium followed by incubation for 48 h at 37°C under anaerobic conditions. Bacterial cells were pelleted at 10,000 × *g*, and the supernatant was sterile-filtered through an 0.2-μm filter and stored at −20°C until used. Supplemented *L. iners* culture supernatant was prepared by adding 2% d(−) lactic acid at pH 4 in a 1:1 ratio to *L*. *iners* culture supernatant prepared as described above. Lactic acid concentrations of the culture supernatants were determined using the EnzyChrom d(−) and l(+) lactate kits (BioAssay Systems) per the manufacturer’s instructions. Absorbance was determined at 565 nm (0 min and 20 min), and provided standards were used to generate a standard curve.

### C. trachomatis strains and EB preparation.

C. trachomatis serovars D and L2 were propagated in HeLa monolayers as previously described in the work of Tan et al. (75). Briefly, the serovars were cultivated in 100-mm^2^ tissue culture dishes in Dulbecco’s modified Eagle’s medium (DMEM) (Mediatech) supplemented with 10% FBS (Sigma-Aldrich) at 37°C in 5% CO_2_. Monolayers were gently rocked for 2 h, fresh medium was added, and the infection was allowed to progress for 48 h. C. trachomatis particles were harvested (stock inclusion-forming units [IFU], 2.11 × 10^8^ and 1.9 × 10^8^ IFU/ml for serovar D and L2, respectively), and stored at −80°C. C. trachomatis serovars were used directly from −80°C stock for all experiments. C. trachomatis was inoculated at a multiplicity of infection (MOI) of 1 in the 3D model and of 2 in the 2D model. To evaluate the effect of C. trachomatis direct exposure to d(−), d/l, or l(+) lactic acids, 2 × 10^5^ C. trachomatis IFU were incubated for 30 min in A2EN culture medium pH adjusted with the different lactic acid solutions described below. We tested the effect of lactic acid isomers on C. trachomatis serovar D, which is commonly associated with cervical infections, and C. trachomatis serovar L2. Similar results were obtained (Fig. S1F). C. trachomatis serovar D has low infectivity toward cultured cells with infection rarely surpassing 10% of host cells. This is in contrast to C. trachomatis serovar L2, in which up to 75% of host cells were infected. For this reason and because similar results were obtained, all reported experiments were carried out using C. trachomatis serovar L2, which ultimately allowed us to observe larger differences under the various conditions tested.

### C. trachomatis infectivity assays.

Each transwell containing mature A2EN 3D model cells was exposed in the apical and basal compartments to 50 μl and 350 μl, respectively, of either culture supernatants from various *Lactobacillus* spp., pH-adjusted culture media [d(−), d/l, or l(+) lactic acid, HCl, at pH 4 or 7], A2EN culture medium, or NYC III medium for 30 min at 37°C. Medium was removed, and cells were rinsed with phosphate-buffered saline (PBS), and then apically incubated with C. trachomatis at a multiplicity of infection (MOI) of 1 for 2 h at room temperature. C. trachomatis was then removed, and cells were incubated for an additional 46 h in fresh medium at 37°C in 5% CO_2_. When required, secondary infection assays were performed and analyzed as detailed below.

Similarly, for coverslips (2D model), cells were exposed to 250 μl of medium or pH-adjusted medium as described above, incubated for 30 min at 37°C, rinsed, and replaced with 100 μl of cell culture medium containing C. trachomatis serovar L2 or serovar D at an MOI of 2. The cells were rocked for 2 h at room temperature, the medium was removed, and fresh medium was added and incubated for an additional 46 h at 37°C in 5% CO_2_.

### (i) Prolonged protection.

3D A2EN cells were exposed to different lactic acid solutions (pH 4), as detailed above, or culture supernatants for 30 min, and cells were rinsed with PBS and then exposed to C. trachomatis at an MOI of 1 immediately (0 h) or 24 h or 48 h later. Experiments were allowed to progress as previously described followed by secondary infection assay analysis.

### (ii) Secondary infection assay.

To assess the level of C. trachomatis infection in the 3D A2EN epithelial cell model, we used a semiquantitative secondary infection assay performed as described by Kessler et al. (76). The number of cell-containing inclusions obtained in this assay is a good surrogate for the level of C. trachomatis infection obtained on the 3D A2EN epithelial cell model, as total numbers of inclusions counted directly in the 3D A2EN model system (Fig. S1C) were proportionally similar to that obtained with the secondary infection assay (Fig. 2). Briefly, 3D A2EN cells previously infected for 48 h were rinsed with PBS, and the collagen was removed from the transwell membrane, which was then excised, placed in water, and passed three times through a 26-gauge syringe. The resultant lysate was diluted 1:5 and immediately used to infect HeLa cells with centrifugation at 900 × *g* for 1 h. Each experiment was controlled and standardized for all parameters, including lysate preparation, amount of lysate used, and time of exposure. The lysate was removed, fresh medium was added, and the cells were incubated for an additional 22 h at 37°C in a 5% CO_2_ atmosphere.

### Immunofluorescence staining and imaging.

Upon completion of the various infection assays, epithelial cells were prepared for confocal (3D model) or fluorescent (2D model) microscopy. For secondary infection and 2D assays, epithelial cells were rinsed with PBS, fixed with 95% methanol (Sigma), and then incubated for 1 h in MicroTrak (chlamydial) stain (Trinity Biotech 8H019UL) at 1:10. The nuclei of epithelial cells were stained using 1:500 Hoechst (Invitrogen). For direct 3D model staining, A2EN cells were rinsed with PBS, fixed with 4% paraformaldehyde (PFA), and permeabilized with 0.25% Triton X-100 followed by treatment with 0.1% Triton X-100 in PBS-fish skin gelatin (FSG) (0.66%) (Sigma-Aldrich). The cells were then stained for chlamydial inclusions with 5 μg/ml of mouse anti-human chlamydia lipopolysaccharide (LPS) (primary antibody [Ab]) (US Biological C4250-51F) for 90 min followed by 200 μg/ml of goat anti-mouse Alexa Fluor 488 (Invitrogen A-11029) for 60 min in the dark. Epithelial cells were imaged by staining the nuclei with 1:500 Hoechst (Invitrogen) for 10 min in the dark. Secondary infection and 2D primary infection epithelial cell culture models on coverslips were imaged using a Zeiss Axio Imager Z1 (Zeiss). Epithelial cells from the 3D primary infection model were imaged using a Zeiss Duo 5 confocal microscope. z-stack slices were created, and three consecutive slices were compressed to create images for analysis.

### Confocal staining.

For direct staining of primary-infected 3D A2EN cells, cells were rinsed with PBS, fixed with 4% paraformaldehyde (PFA) for 30 min, and permeabilized with 0.25% Triton X-100 (Sigma catalog no. T9284; St. Louis, MO) in PBS for 10 min, followed by treatment with 0.1% Triton X-100 in PBS-fish skin gelatin (FSG) (0.66%) (Sigma-Aldrich catalog no. G7765) for 20 min. The cells were then stained for chlamydial inclusions with 5 μg/ml of mouse anti-human chlamydia LPS (primary Ab) (US Biological catalog no. C4250-51F; Salem, MA) in Triton X-100–PBS–FSG solution for 90 min. Secondary antibody staining was done by adding 200 μg/ml of goat anti-mouse Alexa Fluor 488 (Invitrogen catalog no. A-11029; Carlsbad, CA), in Triton X-100–PBS–FSG solution and incubated for 60 min in the dark.

### Image and statistical analyses.

For secondary infection assays and primary infection 2D epithelial cell culture models, the number of inclusions, total number of host cells, and number of host cells containing inclusions were determined using the Cell Profiler imaging software (77), from scoring 5 images. The percentage of infected cells was determined per condition, and the percentage of total number of inclusions was analyzed based on either medium only or pH 7 inclusions used as control. All graphs were created using the GraphPad Prism software (GraphPad Software). 3D images created from compressing confocal slices were opened using the ImageJ program (NIH). Using the cell counter plug-in, the total number of cells (blue) and number of inclusions (green) were manually counted and the percentage of primary-infected host cells was determined and graphed. Using the number of pH 7 inclusions as control, the percentage of total inclusions at the various pHs for each condition was analyzed and then graphed. All graphs were created using GraphPad Prism. Statistical analyses were performed within the GraphPad software with both Student’s *t* test and analysis of variance (ANOVA). Differences between conditions were considered statistically significant if the *P* value was ≤0.05.

### Cell proliferation assay.

Cell proliferation was assessed by adding 10 μM EdU (5-ethynyl-2′-deoxyuridine) to culture supernatants and then to starved cells, which were grown on coverslips at the 0-h time point. Cells were fixed using 100% methanol at the end time point, washed with 3% bovine serum albumin (BSA), permeabilized using 0.5% Triton X-100 in PBS, washed with 3% BSA, and stained for 15 min with Alexa 488 mixture per the manufacturer’s instructions (ThermoFisher catalog no. C10337). Cell nuclei were stained for 15 min using Hoechst 33342 at a 1:1,000 dilution in PBS and then rinsed once in PBS before being imaged at 100× using a Zeiss Axio Imager Z1 (Zeiss, Oberkochen, Germany) fluorescence microscope and the green fluorescent protein (GFP) (EdU) or 4′,6-diamidino-2-phenylindole (DAPI) (Hoechst) filters. The amount of DNA synthesis was calculated using CellProfiler (version 2.2.0 rev 9969f42 [77]) by counting the number of green nuclei (EdU stained) relative to blue nuclei (DAPI stained) in five fields per duplicate coverslip. A two-tailed *t* test was applied to test whether differences between means of each scratch assay and EdU experimental conditions were equal to 0, with *P* < 0.05 considered significant (see https://github.com/ravel-lab/smith_thesis_2017/tree/feature/manuscript-2.0/AnalysisPipeline/Scripts for script).

### C. trachomatis infection and inhibition of cell proliferation.

A2EN human cervical epithelial cells were seeded at 1 × 10^5^ on coverslips, grown overnight, and then exposed to starvation medium (EpiLife medium) for 18 h. Coverslips were exposed to either EpiLife complete medium (control) or CDK4 inhibitor CAS 546102-60-7 at 400 nM (Millipore 219476) or Fascaplysin at 350 nM (Millipore 341251) in complete medium. Concurrently, C. trachomatis serovar L2 was added at an MOI of 2 to each coverslip and rocked for 2 h at room temperature. A2EN cells were then exposed to complete medium or inhibitors in the presence of EdU (1 nM) as described above for an additional 22 h at 37°C in 5% CO_2_. Cells were fixed, stained, and imaged for C. trachomatis, EdU (Alexa Fluor 555; ThermoFisher C10638), or cell viability staining as outlined above. The proportion of infected A2EN cells and new DNA synthesis were determined using manual counting and CellProfiler software. A two-tailed *t* test was applied to test whether differences between means of each EdU or infection experimental conditions were equal to 0, with *P* < 0.05 considered significant. A linear model was fitted to mean percent cells stained with EdU versus the mean proportion of infected A2EN cells using the R *lm* package (version 3.2.3).

### Other methods.

Standard protocols, including medium and solution preparation, metataxonomic analysis, small RNA-seq library preparation and sequencing, RNA-seq library preparation and sequencing, miRNA qPCR, bioinformatic analyses, mucus penetration assay, scratch migration assays, cell cyclin D1 Western blotting, and cell transfection are described in Text S1.

### Data availability.

Metataxonomic data sets analyzed during this current study are available in SRA under PRJNA208535. The code used to analyze data and generate tables and figures in the current study, a table showing the EdgeR differential expression results, and a table of ribo-reduced RNA-seq raw read counts are available at GitHub (https://github.com/ravel-lab/smith_thesis_2017/tree/feature/manuscript-2.0).

10.1128/mBio.01548-19.9 TABLE S3 Ribo-reduced RNA-seq library preparation, sequencing, and alignment statistics. Download Table S3, XLSX file, 0.01 MB. Copyright © 2019 Edwards et al. 2019 Edwards et al. This content is distributed under the terms of the Creative Commons Attribution 4.0 International license.
