# Supplementary material for: The Cervicovaginal Microbiota-Host Interaction Modulates Chlamydia trachomatis Infection
Source: mBio. 2019 Aug 6;10(4):e01548-19. doi: 10.1128/mBio.01548-19 (PMC6692509; doi:10.1128/mBio.01548-19)
Supplement: FIG S1 [file mBio.01548-19-sf001.pdf]

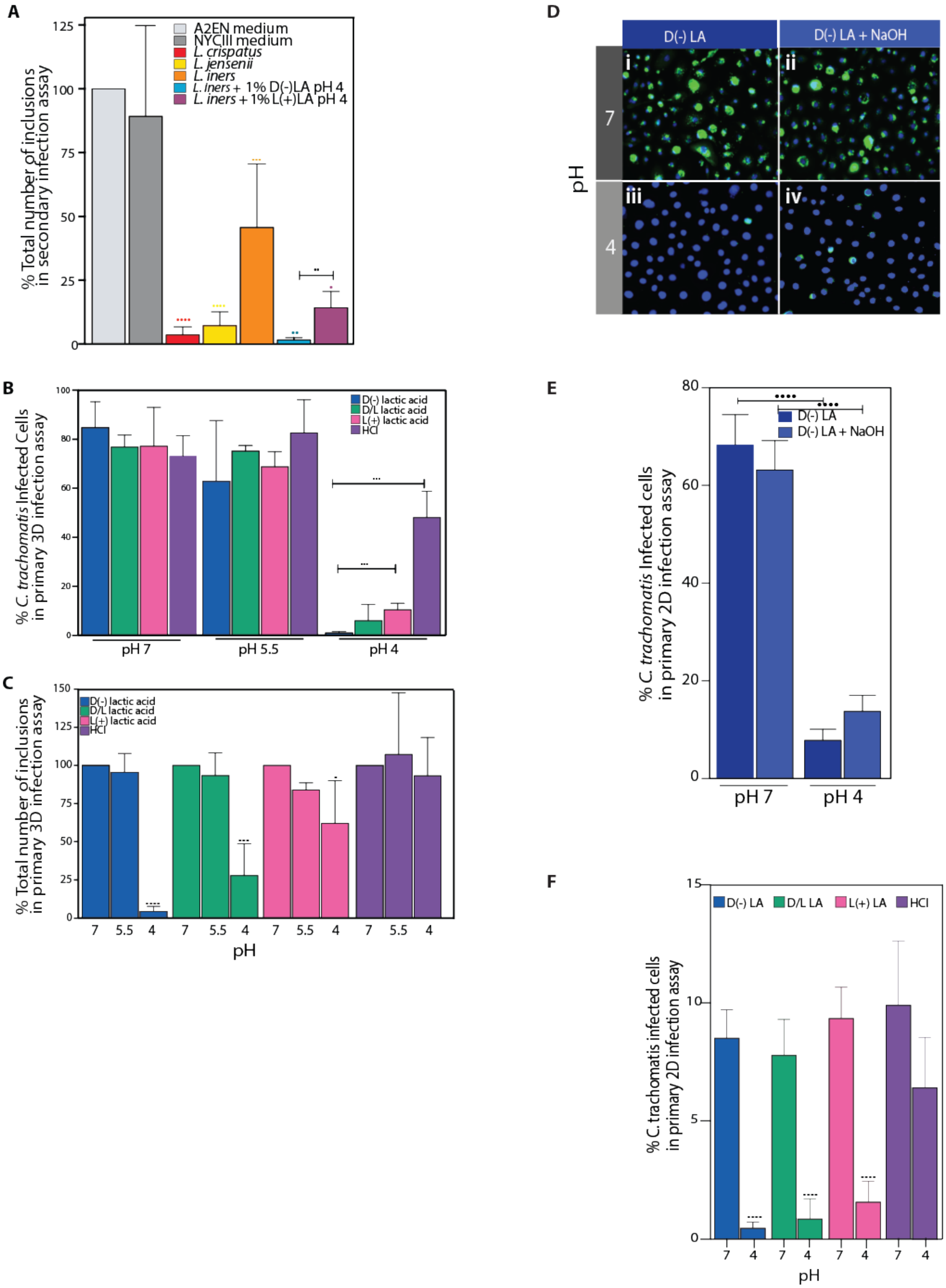
A
% Total number of inclusions in secondary infection assay
A2EN medium
NYCIII medium
L. crispatus
L. jensenii
L. iners
L. iners + 1% D(-)LA pH 4
L. iners + 1% L(+)LA pH 4
B
% C. trachomatis Infected Cells in primary 3D infection assay
D(-) lactic acid
D/L lactic acid
L(+) lactic acid
HCl
pH 7
pH 5.5
pH 4
C
% Total number of inclusions in primary 3D infection assay
D(-) lactic acid
D/L lactic acid
L(+) lactic acid
HCl
pH
D
D(-) LA
D(-) LA + NaOH
pH
7
4
i
ii
iii
iv
E
% C. trachomatis Infected cells in primary 2D infection assay
D(-) LA
D(-) LA + NaOH
pH 7
pH 4
F
% C. trachomatis infected cells in primary 2D infection assay
D(-) LA
D/L LA
L(+) LA
HCl
pH

G

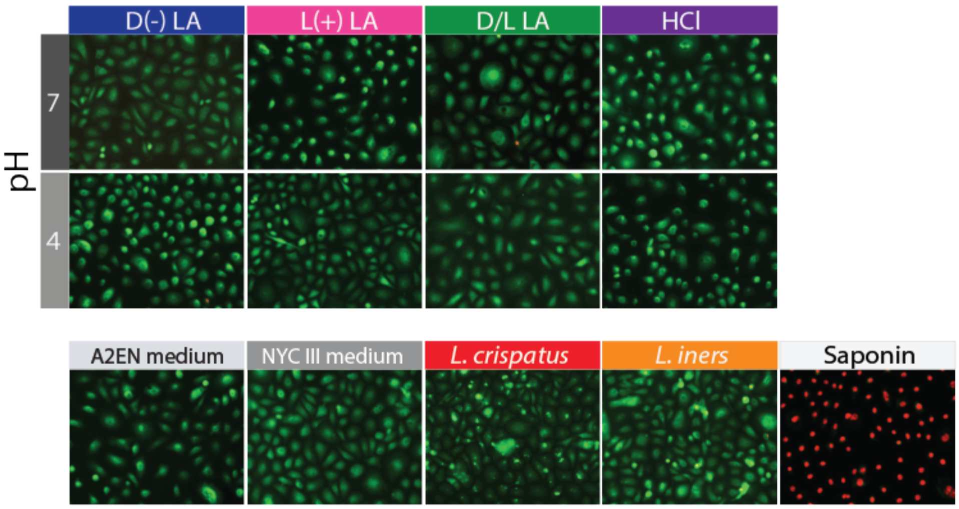
D(-) LA
L(+) LA
D/L LA
HCl
pH
7
4
A2EN medium
NYC III medium
L. crispatus
L. iners
Saponin

H

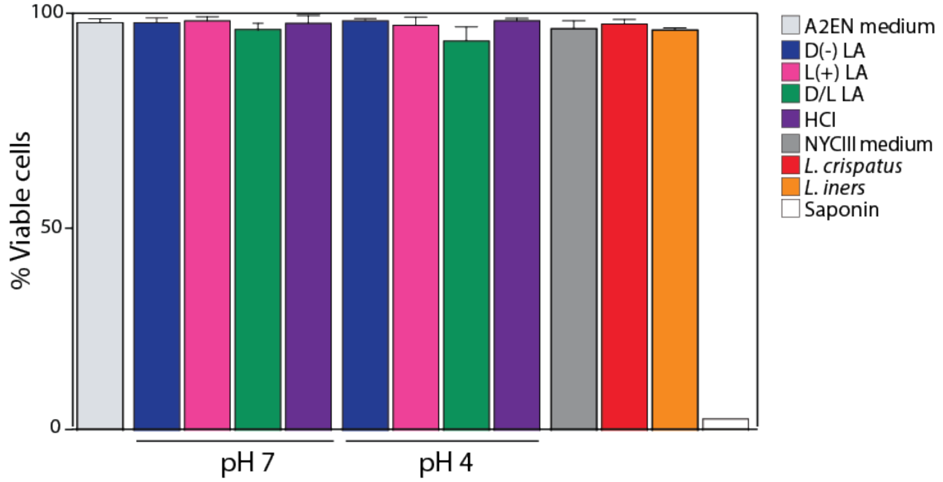
100
50
0
% Viable cells
pH 7
pH 4
A2EN medium
D(-) LA
L(+) LA
D/L LA
HCl
NYCIII medium
L. crispatus
L. iners
Saponin

I

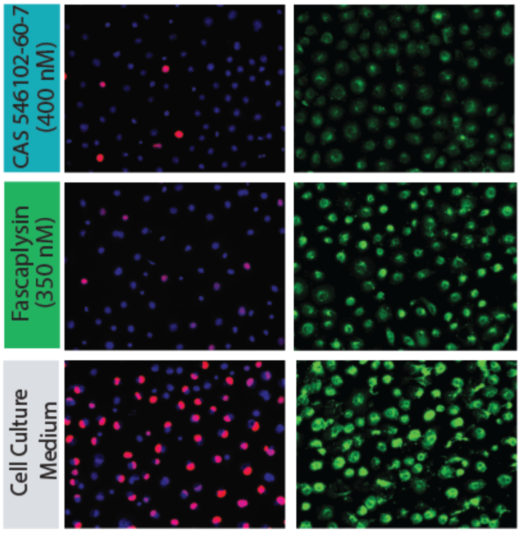
CAS 546102-60-7
(400 nM)
Fascaplysin
(350 nM)
Cell Culture
Medium

Fig. S1. A. Effect of *Lactobacillus* spp. culture supernatants on the total number of inclusions. Post *Lactobacillus* spp. culture supernatant exposure, *C. trachomatis* serovar L2 3D assay infection and subsequent secondary infection assays, the total number of inclusions in the secondary assay were determined using the Cell Profiler software. The percentage of inclusions was determined using the number of inclusions from cells exposed to medium only as control. **B. and C.** Direct effect of lactic acid on *C. trachomatis* infection on the 3D A2EN epithelial cell model. Post exposure to lactic acid and HCl at various pHs, followed by *C. trachomatis* serovar L2 infection, the percentage of infected host cells (**B**) was determined. The total number of inclusions were manually counted, and the percentage of inclusions determined using the number of inclusions at pH7 for each condition as control (**C**). **D. and E**. Effect of pH driven by increasing the concentration of D(-) lactic acid or by maintaining the concentration of D(-) lactic acid and adjusting pH with NaOH. A monolayer of A2EN cells was exposed to D(-) lactic acid for 30 min (A). Lactic acid was either prepared at 15mM (pH 7) and 28mM (pH 4) (**D.i** and **iii**), or a 1% D(-) lactic acid solution was prepared and pH adjusted with 1M NaOH to produce pH 7 and 4 (**D.ii** and **D.iv**). *C. trachomatis* was added at MOI of 2 for 2h, removed and the cells incubated for an additional 46h, then fixed, permeabilized, stained and chlamydial inclusions visualized via fluorescent microscopy. Shown are representative images (**D.i-iv**). The number of inclusions (green) and host nuclei (blue) were determined using the Cell Profiler imaging software and the percentage of infected epithelial cells quantified (**E**) from two independent experiments. **F.** Effect of D(-), D/L racemic mixture, L(+) lactic acid on *C. trachomatis* serovar D infectivity. A 2D model of A2EN cells was exposed to D(-), D/L racemic mixture, L(+) lactic acid or HCl at pH 7 or 4 for 30 min. Cells were washed, fresh medium added and *C. trachomatis* serovar D added at a ~MOI 20 for 2h followed by an additional 46h incubation. The number of inclusions and host nuclei were determined using the Cell Profiler imaging software and the percentage of infected epithelial cells determined from at least two independent experiments. **G. H. and I.** A2EN epithelial cell viability post-incubation with lactic acid at pH 4 or pH 7, *Lactobacillus* spp. culture supernatants or cyclin D inhibitors. A2EN epithelial cells were incubated for 30 min with either cell culture media, D(-), D/L racemic mixture, L(+) lactic acid at pH 4 or pH 7, NYC III bacterial culture medium, culture supernatant from *L. crispatus*, *L. iners* or 0.5% saponin for 10 min. Epithelial cells were then treated with 4μM Calcein AM and 1μM EthDIII for 1h and imaged via fluorescence microscopy. Representative images are shown (**G**). The number of viable (green) and dead (red) cells, were determined manually and the percentage of live host cells quantified from three independent experiments (**H**). Proliferation of A2EN cells after exposure to EpiLife complete medium (control), CDK4 inhibitor CAS 546102-60-7 (400nM) or Fascaplysin (350nM) was determined by staining for new DNA synthesis (EdU nucleobases positive staining - red) and for A2EN nucleus (Hoechst staining - blue) (left column). Viability of these cells were evaluated by treatment with 4μM Calcein AM and 1 μM EthDIII for 1h. Both set of experiments were imaged via fluorescence microscopy. Representative images are shown (**I**). Statistical significance is shown as • p-value <0.01, ••• p-value <0.001 and •••• p-value<0.0001.
