# Supplementary material for: The Cervicovaginal Microbiota-Host Interaction Modulates Chlamydia trachomatis Infection
Source: mBio. 2019 Aug 6;10(4):e01548-19. doi: 10.1128/mBio.01548-19 (PMC6692509; doi:10.1128/mBio.01548-19)
Supplement: FIG S2 [file mBio.01548-19-sf002.pdf]

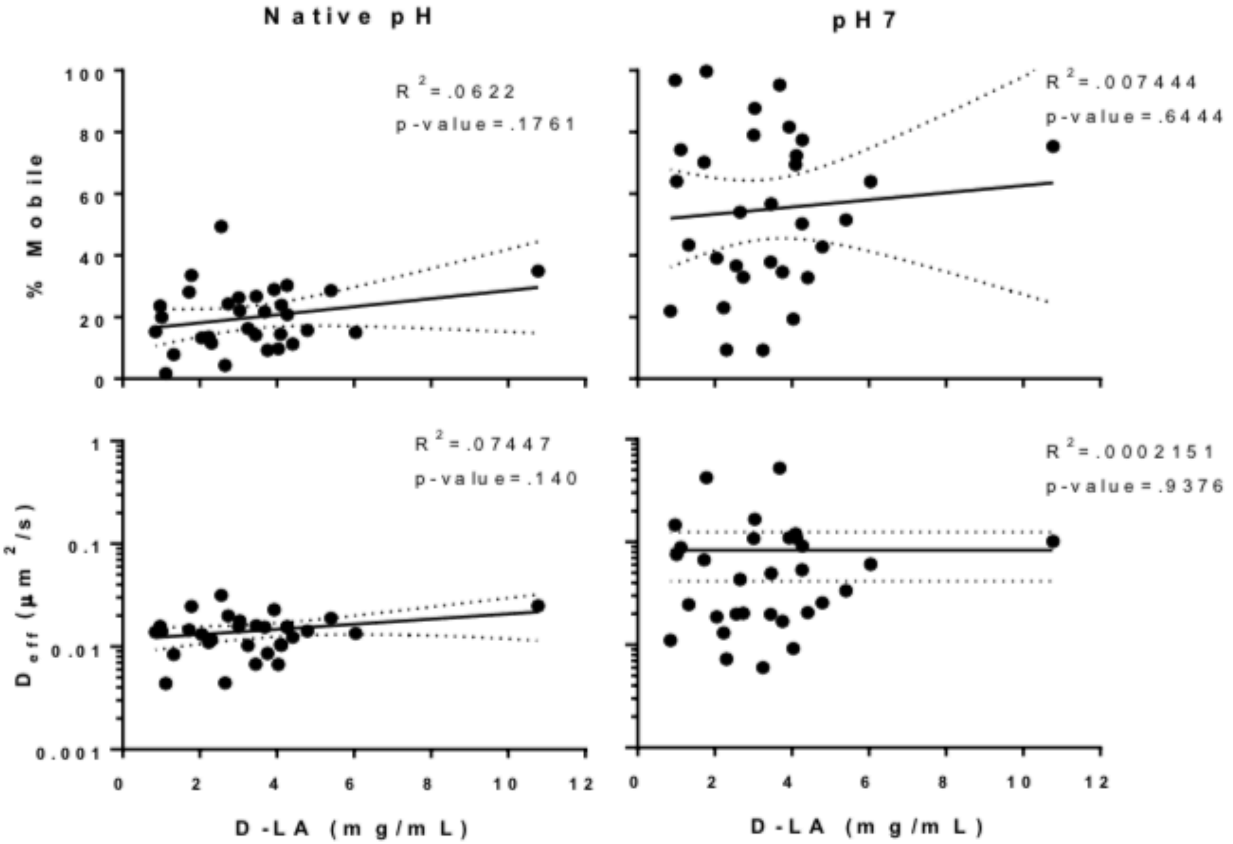

Fig. S2. Mobility of fluorescently labelled CT serovar L2 in native and pH neutralized cervicovaginal mucus (CVM) compared to D-lactic acid concentrations. CVM samples from 34 reproductive-aged women were analyzed for lactic acid measurements by diluting aliquots of whole CVM 1:5 (w/w) with PBS and centrifuging to obtain cell-free supernatant, which was assayed for D-lactic acid using a D/L-lactic acid kit (R-Biopharm) adapted to a 96-well format. Diffusivity and mobility of fluorescently labelled CT serovar L2 were quantified using fluorescent microscopy and multiple particle tracking. Data represent geometric average effective diffusivities (D_{eff}) at a timescale of 0.2667 seconds and fraction of mobile particles corresponding to particles with displacements of less than approximately 200nm at that timescale. Solid lines represent the line of best fit for either linear regression (% mobile) or semi-log regression (D_{eff}). Dotted lines represent the 95% confidence interval around the best-fit line.
