# Supplementary material for: The Cervicovaginal Microbiota-Host Interaction Modulates Chlamydia trachomatis Infection
Source: mBio. 2019 Aug 6;10(4):e01548-19. doi: 10.1128/mBio.01548-19 (PMC6692509; doi:10.1128/mBio.01548-19)
Supplement: TEXT S1 [file mBio.01548-19-s0001.pdf]

SUPPLEMENTARY MATERIALS

The cervicovaginal microbiota-host interaction modulates *Chlamydia trachomatis* infection

Vonetta L. Edwards, Steven B. Smith, Elias J. McComb, Jeanne Tamarelle, Bing Ma, Michael S. Humphrys, Pawel Gajer, Kathleen Gwilliam, Alison M. Schaefer, Samuel K. Lai, Mishka Terplan, Katrina S. Mark, Rebecca M. Brotman, Larry J. Forney, Patrik Bavoil, Jacques Ravel

SUPPLEMENTARY MATERIALS AND METHODS

Media. *NYCIII medium*: 10 g/L proteose peptone, 10 g/l beef extract, 5 g/l yeast extract, 5 g/L NaCl, 1.2 g/L $MgSO_4$, 2 g/L $MnSO_4.H_2O$, 5.7 g/L K_2HPO_4, 20 g/L glucose, 10% FBS. *TSB medium:* 17g/L tryptone (Pancreatic Digest of Casein), 3g/L Soytone (Peptic Digest of Soybean), 2.5g/L glucose, 5g/L NaCl 2.5 g/L K_2HPO_4. *EpiLife complete medium:* EpiLife Media (GIBCO M-EPI-500-CA) supplemented with EDGS EpiLife Defined Growth Supplement (GIBCO S-012-5) and L-glutamine (25030156), (GIBCO, Gaithersburg, MD). *VK2 complete medium:* Keratinocyte SFM, (ThermoFisher # 17005042, Waltham, MA), with bovine pituitary extract (0.05 mg/ml), epidermal growth factor (0.1 ng/ml) and $CaCl_2$ (0.4 mM).

Vaginal sample collection. *CHARM:* Adolescents and young adults positive for *C. trachomatis* infection were screened at point-of-care centers and community-based outreach sites and upon notice of positive *C. trachomatis* nucleic acid amplification tests (NAATs) eligible participants were extended the offer to enroll in the study. Participants provided 150 baseline CT-positive samples – Visit 1 (V1), prior to antibiotic treatment with 1 mg single dose azithromycin, and follow-up samples 3 months post-treatment Visit 2 (V2), (94 samples). Women in this cohort were compared with a control (C) group of 99 self-reported healthy African-American women (13 to 45 years old), from a previous study (1). *SENTINEL:* Reproductive age (18-44 years old) women who were self-reported not pregnant nor had any visible spotting in the previous 48h; self-collected cervicovaginal mucus samples using a menstrual cup collection device (Instead Softcup) and placed them in a 50 ml conical tube. The samples were spun off the Softcup and aliquoted for 16S rRNA gene sequencing, lactic acid concentration determination and fluorescent CT penetration analysis. Participants also provided a urine sample after cervicovaginal mucus collection for chlamydial and gonococcal nucleic acid amplification testing (NAATs). *UMB-HMP:* A cohort of 135 reproductive-age non-pregnant women, recruited at the University of Alabama at Birmingham sampled their vagina daily for 10 weeks (2). Swabs were stored at -80°C in 2 ml of Amies Transport Media/RNAlater (50%/50% v/v) (Qiagen). Subjects answered daily questionnaires on sexual activity, menstruation and symptomatology among others.

DNA extraction, and 16S rRNA gene amplification and sequencing. Whole genomic DNA was extracted from a 1ml aliquot of re-suspended Amies Eswab solution using previously

reported procedures (1). Briefly, mid-vaginal Eswabs, stored in Amies transport medium (Copan) were thawed on ice and vortexed briefly. A 0.5 ml aliquot of the cell suspension was transferred to a FastPrep Lysing Matrix B (MP Biomedicals) tube containing 0.5 ml of PBS (Invitrogen). A cell lysis solution containing 5 µl lysozyme (10 mg/ml, EMD Chemicals), 13 µl mutanolysin (11,700 U/ml, Sigma-Aldrich), and 3.2 µl lysostaphin (1 mg/ml, Ambi Products) was added and samples were incubated at 37°C for 30 min. Then, 10 µl Proteinase K (20mg/ml, Invitrogen), 50 µl 10% SDS (Sigma-Aldrich), and 2 µl RNase A (10 mg/ml, Invitrogen) were added and samples were incubated at 55°C for 45 min. Cell lysis by mechanical disruption was performed on a FastPrep homogenizer at 6 m/s for 40 s, and the lysate was centrifuged on a Zymo Spin IV column at 7000 x *g* for 1 min. (Zymo Research). Lysates were further processed on the QIAsymphony platform using the QS DSP Virus/Pathogen Midi Kit (Qiagen) according to the manufacturer's recommendation. DNA quantification was carried out using the Quant-iT PicoGreen dsDNA assay (Invitrogen). Sequencing libraries were constructed using either 1 step or 2 step PCR (details in supplementary methods) (3, 4). Composition of the vaginal microbiota was assessed by metataxonomics and amplicon sequencing of the 16S rRNA gene V3-V4 hypervariable regions (5, 6). Libraries were sequenced on an Illumina MiSeq instrument using 600 cycles generating paired-end 300 bp sequence reads. The sequences were de-multiplexed using a dual-indexing approach (3).

1-Step PCR library construction. Primer sequences ranged from 90-97 bp and amplification was performed using Phusion Taq Master Mix (1X, ThermoFisher) with 3% DMSO, 0.4 mM of each primer, and 5 mL of genomic DNA. A standard volume of genomic DNA was used for each library. Cycling conditions were as follows: initial denaturation at 98°C for 30s, 30 cycles of denaturation at 98°C for 15s, annealing at 58°C for 15s, and elongation at 72°C for 15s, followed by a final elongation step at 72°C for 60s (3).

2-Step PCR library construction. This library preparation (4) is modified from an Illumina protocol (https://support.illumina.com/downloads/16s_metagenomic_sequencing_library_preparation.html). The 16S rRNA gene V3-V4 region from genomic DNA was targeted using bacterial primers 338F and 806R combined with a heterogeneity spacer of 0-7 bp, and Illumina Sequencing Primers. A single PCR master mix containing an equal ratio of all primers, was used. Each PCR reaction contained 1X Phusion Taq Master Mix (ThermoFisher), Step 1 Forward and Reverse

primers (0.4 mM each), 3% DMSO, and 5 mL of genomic DNA. The following cycling conditions were used for PCR amplification: initial denaturation at 94°C for 3 min, 20 cycles of denaturation at 94°C for 30s, annealing at 58°C for 30s, and elongation at 72°C for 60s, and a final elongation step at 72°C for 7 min. The resultant amplicons were diluted 1:20, and 1 mL was used in the second step PCR which introduced an 8 bp dual-index barcode to the 16S rRNA gene amplicons and the flow cell linker adaptors using primers containing a sequence that anneals to the Illumina sequencing primer sequence introduced in step 1. Each primer was added to a final concentration of 0.4 mM in each sample specific reaction, along with Phusion Taq Master Mix (1X) and 3% DMSO. Phusion Taq Polymerase (ThermoFisher) was used with the following cycling conditions: initial denaturation at 94°C for 30s, 10 cycles consisting of denaturation at 94°C for 30s, annealing at 58°C for 30s, and elongation at 72°C for 60s, followed by a final elongation step at 72°C for 5 min.

Microbiota bioinformatic analysis. The resulting forward and reverse sequence fastq files were split by sample using the QIIME-dependent script split_sequence_file_on_sample_ids.py, and primer sequences were removed using TagCleaner (version 0.16) (7). Further processing followed the DADA2 Workflow for Big Data and dada2 (v. 1.5.2) (https://benjjneb.github.io/dada2/bigdata.html) (8, 9). Taxonomy was assigned to each read using a novel fifth-order Markov Chain Monte Carlo taxonomic classifier (available at http://ravel-lab.org/speciateit) and taxa frequencies normalized to total per-sample read counts. Community state types (CSTs) were identified by calculating the Jensen-Shannon divergence among samples, followed by hierarchical clustering with complete linkage (1). Clusters were assigned to one of the five CSTs described previously (1, 5).

Antimicrobial resistance test. Minimal inhibitory concentration (MIC) for 22 vaginal bacteria strains was determined by broth microdilution using azithromycin and doxycycline in TSB and NYCIII medium (Sigma-Aldrich). Using a previously published protocol, bacteria were exposed to 12 dilutions ranging from 0.03-640 mg/L and the inhibitory concentration determined by OD(10).

Lactic acid solutions pH adjusted by increasing lactic acid concentration. D(-), D/L (racemic mixture of each isomer) or L(+) lactic acids (Sigma-Aldrich) were added to A2EN complete medium at concentrations of 15 mM, 22.5 mM and 28 mM. pH was measured and when necessary adjusted to pH values of 7, 5.5 and 4, respectively using 100mM lactic acid. Hydrochloric acid (HCl) (Sigma) was added at concentrations of 15 mM, 17 mM and 19 mM, and adjusted to pH 7, 5.5 and 4, respectively using 100 mM HCl.

Lactic acid solutions pH adjusted from a 1% lactic acid with NaOH. A 30% (w/v) stock solutions of each D(-), D/L or L(+) lactic acid were made and used to prepare 1% lactic acid in A2EN complete medium (pH ~3) for each experiment. The pH of the media was adjusted to pH 7 and 4 using 1M NaOH. A 1% (w/v) stock solution of HCl was made, followed by a 30X dilution in A2EN complete medium (pH~3) and adjusted to pH 7 and 4 using 1M NaOH.

A2EN and VK2 cell viability assay. A2EN and VK2 cell viability was performed using the Viability/Cytotoxicity Assay Kit for Animal Live and Dead cells (Biotium) as per the manufacturer's instructions. Briefly, epithelial cells were either exposed to media containing lactic acid or to different culture supernatants for 30 min or to 20% diluted culture supernatants for up to 24h, rinsed and incubated with 4 µM Calcein AM and 1 µM EthDIII for 1 h in the dark. Images were taken using the FITC and RFP filters on a Zeiss Axio Imager Z1(Zeiss). Manual analysis was performed to determine the percentage of live (green) relative to dead (red) cells within each sample.

Mucus penetration imaging. mCherry labeled *Chlamydia trachomatis* serovar L2 were exposed to the cervicovaginal mucus and analyzed in a manner similar to fluorescent HIV (11). Briefly fluorescent bacteria or beads were added at 5% (vol/vol) to 20µl of CVM placed in a custom-made glass chamber and incubated for 1 h at 37°C prior to microscopy. An aliquot of CVM was titrated to pH 6.8 to 7.1 using small volumes (~3% [vol/vol]) of 3 N NaOH and then analyzed. Using an electron multiplying charge-coupled-device (EMCCD) camera (Evolve 512; Photometrics, Tucson, AZ) mounted on an inverted epifluorescence microscope (AxioObserver D1; Zeiss, Oberkochen, Germany) the translational motions of the particles were recorded. Using MetaMorph imaging software (Molecular Devices, San Jose, CA) videos (512 X 512 pixels, 16-

bit image depth) were captured and the tracking resolution was determined by tracking the displacements of particles. Trajectories of ≥40 particles per frame on average were analyzed for each sample, typically corresponding to >100 individual particle traces throughout the video.

Sample selection for small RNA-sequencing.

Samples for small RNA-seq were selected from vaginal microbiota profiles previously characterized by metataxonomics analysis (16S rRNA gene sequencing) (2). Samples were self-collected daily for 10 weeks by 135 reproductive-age women and dropped off weekly to the study site. A clinical visit was performed at enrollment, week 5 and week 10 of the study. Sample selection aimed at representing a diversity of vaginal microbiota types and samples were selected from participants with vaginal microbiota longitudinal profiles: 1) persistently dominated by *Lactobacillus* spp. with few or no reported vaginal symptoms and no diagnosis of BV based on Amsel criteria at all three clinical visits during the study period (5 subjects); 2) with persistent Nugent-BV-associated community state type (CST IV) that were sometimes accompanied by vaginal symptoms, and the participant was Amsel-BV positive for at least one of the three clinical visits during the study period (5 subjects); and 3) with at least one transition between *Lactobacillus* spp. dominance and Nugent-BV associated CST IV (6 subjects). A total of 83 samples were selected from 16 subjects (Nugent score, CST, and subject characteristics are shown in Table S1B).

Total RNA extraction. Total RNA extraction from vaginal swabs was performed in random order on selected samples across multiple days and subjects to minimize batch effects. The MasterPure™ Complete DNA and RNA Purification Kit (Epicentre) was used to extract total RNA from a 250 µl aliquot of swab suspension stored in Amies transport medium containing RNAlater or confluent adherent VK2 epithelial cells following the manufacturer's recommendations. DNA was removed by two consecutive additions of 1 µl TURBO DNA-free DNase with 30 min incubations at 37°C (Life Technologies) and DNAse inactivation as per manufacturer's recommendations. RNA quality and quantity were measured using Agilent TapeStation and RNA screen tape (Agilent 5067-5576). Total RNA was stored at -80°C until further use. All samples yielded at least 20 ng total RNA.

Small RNA sequencing - library construction. Small RNA-seq libraries were prepared using the TruSeq Small RNA kit per manufacturer's recommendations (Illumina #RS-200-0012, San Diego, CA), with CleanTag Ligation from TriLink Biotechnologies' modifications (TriLink Biotechnologies #L-3203, San Diego, CA) at 1:3 5' and 3' adaptor dilutions and 15 cycles for library enrichment. Both the 3' CleanTag Adaptor and 5' Adaptor were diluted 1:3 in nuclease-free water to accommodate 50-100 ng total RNA input. The RNA template was denatured for 2 min at 70°C, then 1µl diluted 3' adaptor, 1µl RNase Inhibitor, 1µl Enzyme 1 and 5µl Buffer 1 were added to the template, mixed, and incubated at 28°C for 1 hour followed by incubation at 65°C for 20 min. Following this step, 4µl nuclease-free water, 1µl Buffer 2, 1µl RNase Inhibitor, and 2µl Enzyme 2, was added to the RNA template and the 3' adaptor mixture. The diluted 5' adaptor was denatured for 2 min at 70°C, then 2µl was added to the mixture and incubated at 28°C for 1h followed by an incubation at 65°C for 20 min. The tagged library underwent reverse transcription by adding 2µl RT primer (TruSeq kit), 1.92µl RNAse-free water, 5.76µl RT buffer (SuperScript II/Life Technologies, Carlsbad, CA), 1.44µl dNTPs, 2.88µl 0.1 mM DTT, 1µl RNAse Inhibitor, 1µl superScript II (Life Technologies, Carlsbad, CA), and then incubated at 50°C for 1h. The cDNA was PCR enriched by adding 40µl 2X Phusion High Fidelity Taq Polymerase Mastermix (ThermoFisher), and 2µl each of the universal forward primer and a sample-specific index, then PCR amplified using the following conditions: 98°C for 30s, [15 cycles of 98°C for 10s, 60°C for 30s, 72°C for 15s] and a final extension at 72°C for 10 min. The enriched libraries were purified using Agencourt AMPure XP beads (Beckman Coulter, Brea, CA) by adding 80µl beads to the 80µl reaction volume and incubating for 10 min to bind DNA. The beads were magnetized for 4 min, then the supernatant containing the library was transferred to a new tube where 144µl beads were added and incubated for 10 min to bind DNA. The beads were magnetized again for 4 min, the supernatant discarded, and the beads washed twice with 500µl 70% ethanol. After the wash, the beads were left to air-dry before resuspending in 17µl nuclease-free water for 2 min. The solution was re-magnetized and 15µl was transferred as the small RNA-seq library.

Small RNA sequencing – sequencing, alignment, quality control and read mapping. RNA Integrity (RIN) values from vaginal swabs were of lower quality for full-length transcript RNA-seq, however, miRNAs were sufficient for analysis as evidenced by lack of correlation between

miRNA reads and RINe (Fig. S3A and S3B). Small RNA-seq libraries were sequenced on a HiSeq 4000 with either 75 bp single-end (SE) or 150 bp paired-end (PE) reads, at about 20-40 million reads per library (approximately 10% sample per lane). Reads from the R1 fastq file (150 PE) or R2 fastq file (75 SE) were trimmed using Trimmomatic-0.33 with the following parameters: LEADING:3, TRAILING:3, SLIDINGWINDOW:4:15 and MINLEN:16, then visualized for quality using fastqc (version 0.10.0). Reads were aligned with bwa (version 0.5.9-r16) using 2 as the maximum number of mismatches between reference and read to the following series of references, in which all unaligned reads were aligned to the next in series: first, all tRNA from the GtRNAdb (http://lowelab.ucsc.edu/GtRNAdb/) (12), then human rRNA (hum5SrDNA and humRibosomal each a part of Illumina's iGenomes available at ftp://illumina.com/Homo_sapiens/UCSC/hg19/Homo_sapiens_UCSC_hg19.tar.gz, downloaded August 17, 2015), *G. vaginalis* ATTC 14019 (NCBI reference NC_014644.1) (13), *L. iners* ATCC 55195 (NCBI reference NZ_GL622333.1), and finally human hg19 (also downloaded from Illumina's iGenomes as above). Reads aligning to human miRNA regions were annotated using HTSeq (version 0.5.3p3, Python version 2.7) and annotations from primary transcript miRNAs from the miRBase v20 (GTF version 3, GRCh37.p5, NCBI Assembly GCA 000001405.6).

Ribosomal RNA-depleted (rRNA-depleted) RNA sequencing - library construction. All reagents were obtained from Illumina unless otherwise stated. All rRNA-depleted RNA-seq libraries were carried out using the TruSeq Ribo-Zero Stranded Total RNA kit per manufacturer's recommendations (Illumina # RS-122-2203) using 10µl of total RNA as extracted in methods. For each sample, 5µl of rRNA binding buffer and 5µl Ribo-Zero human/mouse/rat rRNA removal mix were added and then incubated at 68°C for 5 min. Following this step, the entire volume was added to 35µl rRNA removal beads, incubated for 1 min, and then beads were captured on a magnetic plate. The supernatant was mixed with 99µl RNAClean XP beads (Beckman Coulter #A63987), incubated for 15 min, then placed on a magnetic plate where the supernatant was removed, and beads were washed once with 70% ethanol. Beads were left to dry for 15 min, and 11µl elution buffer was added. To elute the rRNA-depleted RNA, the beads were magnetized, and the supernatant removed by pipetting. An 8.5µl aliquot of the supernatant was added to 8.5µl of the Elute, Prime, Fragment High mix and

incubated at 94°C for 8 min, then placed on ice. First strand cDNA was performed by adding 8µl of previously prepared reverse transcriptase mix (50µl Superscript II (Life Technologies) into 450µl First Strand Synthesis Actinomycete D to the 17µl of rRNA-depleted RNA. The mixture was incubated at 25°C for 10 min, 42°C for 15 min, and then 70°C for 15 min before placing on ice. Second strand synthesis was carried out by adding 20µl Second Strand Marking Master Mix to the mixture and incubating at 16°C for 1h. The reaction was cleaned using 90µl of AMPure XP beads (Beckman Coulter #A63882, Brea, CA), incubated for 15 min, then placed on magnetic plate and the supernatant discarded. The beads were cleaned using two washes with 80% ethanol, then air-dried for 15 min before adding 17.5µl resuspension buffer. The beads were magnetized, and the solution transferred to a fresh tube. To make the fragments compatible with adapters and prevent self-ligation, a 3'-adenosine overhang was added by adding 12.5µl A-Tailing Mix to 15µl of the solution. The mixture was incubated at 37°C for 30 min, then 70°C for 5 min before being transferred to ice. Adapters were ligated by adding 2.5µl Ligation Mix and 2.5µl of a unique dual Illumina index to each sample. The mixture was incubated at 30°C for 10 min and then 5µl Stop Ligation Buffer was added. The ligated cDNA was cleaned using AMPure XP bead clean-up (Beckman Coulter, Brea, CA) by adding 42µl of beads to the mixture, incubating for 15 min, placing mixture on magnetic stand, removing supernatant, and washing twice with 80% ethanol. The beads were resuspended in 52.5µl Resuspension Buffer, (magnetized and 50µl of the supernatant was added to 50µl AMPure XP beads (Beckman Coulter) for 15 min. Beads were magnetized, the supernatant discarded, washed twice with 80% ethanol and dried for 15 minutes. The beads were resuspended in 22.5µl Resuspension Buffer, magnetized, and 20µl of the mixture transferred to a fresh tube for PCR enrichment. To selectively enrich DNA fragments that have adapter molecules on both ends and to amplify the amount of DNA in the library 5µl PCR Primer Cocktail, 25µl PCR Master Mix were added to the mixture and amplification was performed using 15 cycles at 98°C for 10 seconds, 60°C for 30 seconds and 72°C for 30 seconds, and a final extension at 72°C for 5 min. Enriched libraries were cleaned by adding 50µl AMPure XP beads (Beckman Coulter, Brea, CA), incubating for 15 minutes, then placing the tubes on a magnetic stand and discarding the supernatant by pipetting. The beads were washed twice with 80% ethanol, air-dried for 15 min, and resuspended in 32.5µl Resuspension Buffer. The beads were magnetized and 30µl of the supernatant was transferred

for subsequent library validation and sequencing. Libraries fragment size of 200-500bp range were validated on the LabChip GX (PerkinElmer, Waltham, MA).

Ribosomal RNA-depleted (rRNA-depleted) RNA sequencing - quality control and read mapping. RNA-seq libraries were sequenced on an Illumina HiSeq 4000 using the 150 bp paired-end protocol at the Institute for Genome Science's Genomic Resources Center (Baltimore, MD). Indexed RNA-seq libraries were multiplexed at 15 samples per lane. RNA-seq reads were trimmed using *trimmomatic* version 0.33 using the following parameters: ILLUMINACLIP:2:30:10 LEADING:3 TRAILING:3 SLIDINGWINDOW:4:15 MINLEN:36 (using Illumina adapter sequences, remove the first and last 3 bases below quality 3, with a 4 bp sliding window and trimming when quality drops below 15, and dropping reads below 36 bases long) (14). Reads were aligned to the hg19 human genome reference sequence using TopHat v2.1.0 with default parameters (15) (Human Genome Reference hg19 (available through Illumina at iGenomes ftp://illumina.com/Homo_sapiens/UCSC/hg19/Homo_sapiens_UCSC_hg19.tar.gz, downloaded August 17, 2015). Strand-specific genomic feature overlaps were counted using HTSeq version 0.5.3p3 (16) with default parameters (mode=union, minaqual=0, stranded='yes') and the iGenomes annotation as above (Table S3 and https://github.com/ravel-lab/smith_thesis_2017/tree/feature/manuscript-2.0 file Ribo reduced RNA Seq raw read counts.xlsx).

microRNA qPCR. Total RNA samples were converted into polyadenylated cDNA for qPCR analysis by mixing 7µl (50-100 ng) total RNA in water with QIAGEN miScript II RT (QIAGEN # 218160, Venlo, Netherlands) reagents: 2µl Reverse Transcriptase Mix, 2µl 10X Nucleic acids Mix, 4µl 5X miScript HiSpec Buffer and 5µl nuclease-free water. The reaction mixture was incubated at 37°C for 1h, then heated to 95°C for 5 min to stop the reaction per the manufacturer's protocol(17, 18). The resulting cDNA was diluted 1:10 in water before use in the subsequent qPCR assay.

qPCR reactions were performed using 1µl diluted cDNA template, and the miScript SYBR Green PCR Kit by mixing 5µl 1X SYBR Green Mastermix and 1µl each of Universal and miRNA-specific primers in a 10µl reaction volume (QIAGEN #218073 and #218160, miR-

specific primers: MS00031549 (miR-193b-3p, 5'AACUGGCCCUCAAAGUCCCGCU) and MS00033740 (RNU6-2-11) (QIAGEN). qPCR was carried out on a 7900HT thermocycler (ThermoFisher) at the following cycle conditions: 95°C for 15 min, then 40 cycles of: 94°C for 15s, 55°C for 30s and 70°C for 30s. The ΔΔCt method was used to compute the ΔCt between miR-193b and RNU-6 within a sample, and then the ΔCt between *Lactobacillus* spp. CS or lactic acid and cell culture medium (19). The ΔΔCt standard deviation was computed using $\sigma_{\Delta\Delta Ct} = (\sigma,_{\Delta Ct\ \mathit{Lactobacillus}\ spp.}{}^2 + \sigma,_{\Delta Ct\ non\text{-}\mathit{Lactobacillus}\ spp.}{}^2)^{1/2}$, where σ is the standard deviation. A two-tailed t-test was applied to each ΔΔCt, with $p<0.05$ considered significant.

Identification of *Lactobacillus* spp.-associated miRNAs using Random forest. A Random forest model was applied to select miRNAs that best predict *Lactobacillus* spp. relative abundance by utilizing a combination of the R software packages *rfPermute* (version 2.0.1, (20)), *randomForest* (version 4.6-12, (21)) and custom subroutines (available in the R scripts at https://github.com/ravel-lab/smith_thesis_2017/tree/feature/manuscript-2.0/AnalysisPipeline/Scripts). Each sample's *Lactobacillus* spp. relative abundance (proportion of reads mapping to *Lactobacillus crispatus*, *Lactobacillus jensenii*, *Lactobacillus iners*, and *Lactobacillus gasseri*, available in SRA under project PRJNA208535) were used as the response variable to determine the most important miRNAs that predicted the community state while the $\log_2$ transformed normalized miRNA counts were used as predictors. The training set consisted of 70% of available data while model performance was assessed using the remaining 30% of the held-out data. To increase the confidence of feature calls, miRNAs with zero counts in any sample were excluded, as zero miRNA counts could be due to under sampling. Each model underwent 10-fold cross-validation, with 500 permutations to determine the null distribution for p-value calculation. Default parameters for *ntree* (500) and *mtry* (1558 input features/3=519) were used. The algorithm accounted for non-independent samples that originated from the same subject by evenly splitting each cross-fold iteration among subjects using a custom script (available at https://github.com/ravel-lab/smith_thesis_2017/tree/feature/manuscript-2.0/AnalysisPipeline/Scripts). Importance metrics and p-values were calculated based on *rfPermute* and *randomForest* R packages (20, 21). The increase in mean squared error and increase in node purity were used to rank features (22, 23). Statistically significant features were defined as features with p-value<0.05 for any importance metric within a model result.

RNA-seq differential expression and pathway analysis. All analysis scripts can be found at https://github.com/ravel-lab/smith_thesis_2017/tree/feature/manuscript-2.0/AnalysisPipeline/Scripts. Sample replicates were validated by computing the log_2 read count linear correlation coefficients between replicates. Samples with R^2 <0.9 were excluded from further analysis, except where dropping a sample would result in a single sample per time point for a given treatment. To check for contamination, including the presence of human rRNA not aligned to the human reference, the top 10 most abundant unaligned reads per treatment were BLASTed against the non-redundant nucleotide collection to determine any non-human cross-contamination (from experimental sources or within-sequencing lane or human rRNA contamination (24)) Samples having more than 90% human rRNA sequences were excluded from further analysis. Seven of the initial 45 samples either failed sequencing or library construction, had a relatively high proportion (94.3%) of human rRNA reads, or poorly correlated (R^2<0.9) with the other replicate samples and were removed, but each condition still maintained at least duplicate samples for further analysis (Table S3). All of the top 10 most abundant unaligned reads from all samples were of human origin.

The R package *edgeR*, version 3.10.5, was used to compute pairwise differential expression between combinations of each exposure time and BCS vs. cell culture medium (25). Negative binomial dispersion was estimated for samples passing QC by applying the *estimateDisp* function available through *edgeR*. Samples were normalized using the *calcNormFactors* function (26). Reads were fit to a negative binomial generalized linear model using the *glmFit* function available in *edgeR*, using the sample's treatment as the design matrix. Differential expression using *edgeR*'s likelihood ratio test was computed for each gene using the *glmLRT* function. Genes with an average log counts per million (logCPM) >1 , log_2-transformed Fold Change (logFC) > 1 and false discovery rate (FDR) < 0.01 (27, 28)(87) were considered differentially expressed between treatments. The mean logCPM as calculated by *edgeR* is the log_2 counts per million reads, averaged over all the libraries, while log_2FC is the coefficient of the Generalized Linear Model used by *edgeR* (25). Gene expression plots were created using *ggplot2* (version 2.2.1) and custom scripts (29).

Differentially expressed genes for each comparison were used to generate pathway enrichment scores using Ingenuity Pathway Analysis (IPA) (QIAGEN, build version 439932M, content

version 33559992). IPA computes a pathway activation score (z-score) for each pathway comparison based on inferred expression directionality using the logFC of each gene (29).

Determining gene ontology for *Lactobacillus* spp.-associated miRNAs. Experimentally validated miRNA targets were identified using the "strong evidences" list from miRTarBase, Release 6.0 (Sept 15, 2015) (30). The Gene Ontology DIRECT process terms from DAVID (release 6.8, May 2016, (31)) were mapped to experimentally validated miRNA targets. The proportions of targets for each GO DIRECT term were computed for each miRNA. The miRNA with the highest overall expression and most correlated with *Lactobacillus* spp. relative abundance was chosen for further experimentation.

Scratch assay using bacterial culture supernatants. VK2 epithelial cells, seeded at 7.5×10^4 cells/well, were grown to confluence, starved for 24h in base medium at which time a scratch made with a 1 ml sterile pipette tip. VK2 cells were then exposed for up to 22h to either lactic acid medium (0.06% of either D(-) or L(+) lactic acid in VK2 cell culture medium) or culture supernatants (created as previously described) diluted to 20% (v/v) using complete VK2 cell culture medium. Phase contrast images were taken at 100X using a Zeiss Primovert microscope (Zeiss) at 0 and 13h post-culture supernatant exposure. The proportion of cells occupying the scratched area relative to time 0h was used to quantify migration (ImageJ software (version 1.50i, (32)). Total RNA from exposed cells were then extracted by first adding 300 µl RNAlater and mechanically detaching cells from the plate, then using the total RNA extraction method described above. To monitor active DNA synthesis for cell proliferation, EdU (5-ethynyl-2'-deoxyuridine) assay was carried out per manufacturer's instructions (ThermoFisher). Briefly, cell nuclei were stained using Hoechst 33342 (1:1,000) and imaged at 20X using a Zeiss Axio Imager Z1 fluorescence microscope (Zeiss). The amount of DNA synthesis was calculated using CellProfiler (version 2.2.0 rev 9969f42) (33) by counting the number of green nuclei (EdU stained) relative to blue nuclei (DAPI stained) in five fields per duplicate coverslip.

Cell Cyclin D1 (CCND1) Western blot. VK2 epithelial cells were grown and exposed to culture supernatants for 4, 13 or 22 h as described above. For miRNA quantification and scratch assays, medium was removed, total protein extracted using RIPA buffer (Cell Signaling

Technologies 9806S) and cell lysate stored at -80°C. Tris-glycine precast gels, 4-15% (Bio-Rad), were loaded with 20 µg total protein per well and run for 35 min at 140 V before transferring to a PVDF membrane at 20V for 20 min. Membranes were blocked using Odyssey PBS blocking buffer (Li-Cor) for 1 hr. Primary antibodies for purified mouse anti-human Cyclin D1, clone G124-326 (1:300) (BD Pharmingen 554180) and rabbit anti β-actin (1:5,000) (Abcam 8227) were incubated with blocking buffer and 0.2% Tween-20 for 1 hr. Membranes were washed with PBS-0.1%Tween-20 and then incubated with 1:15,000 secondary antibodies (Li-Cor, IRDye 680RD goat anti-rabbit, and IRDye 800CW goat anti-mouse) before a final wash and imaging using an Odyssey® CLx Imager and Image Studio software (version 5.2) (Li-Cor). Protein band intensities were measured using ImageJ Studio software, then CCND1 intensity values were normalized to β-actin loading control value.

Transfection of miR-193b into vaginal epithelial cells. VK2 cells were plated, grown to 80-90% confluency then transfected for 22 hours at 37°C in 5% CO_2 with transfection reagent (DharmaFECT 4 T-2004) and hsa-miR-193b-3p mimic (Dharmacon C-300764-05) or scramble negative control (Dharmacon CN-002000-01) (Dharmacon) at a final concentration of 10nM mimic or scramble. Total protein was extracted as described above.

REFERENCES

1. Ravel J*, et al.* (2011) Vaginal microbiome of reproductive-age women. *Proc Natl Acad Sci U S A* 108 Suppl 1:4680-4687.
2. Ravel J*, et al.* (2013) Daily temporal dynamics of vaginal microbiota before, during and after episodes of bacterial vaginosis. *Microbiome* 1(1):29.
3. Fadrosh DW*, et al.* (2014) An improved dual-indexing approach for multiplexed 16S rRNA gene sequencing on the Illumina MiSeq platform. *Microbiome* 2(1):6.
4. Holm JB*, et al.* (2018) Ultra-high throughput multiplexing and sequencing of >500 bp amplicon regions on the Illumina HiSeq 2500 platform. *Biorxiv*.
5. Gajer P*, et al.* (2012) Temporal dynamics of the human vaginal microbiota. *Sci Transl Med* 4(132):132ra152.
6. Marchesi JR & Ravel J (2015) The vocabulary of microbiome research: a proposal. *Microbiome* 3:31.
7. Schmieder R, Lim YW, Rohwer F, & Edwards R (2010) TagCleaner: Identification and removal of tag sequences from genomic and metagenomic datasets. *Bmc Bioinformatics* 11:341.
8. Girard L*, et al.* (2018) Impact of the griffithsin anti-HIV microbicide and placebo gels on the rectal mucosal proteome and microbiome in non-human primates. *Sci Rep* 8(1):8059.
9. Callahan BJ*, et al.* (2016) DADA2: High-resolution sample inference from Illumina amplicon data. *Nature methods* 13(7):581-583.
10. Wiegand I, Hilpert K, & Hancock RE (2008) Agar and broth dilution methods to determine the minimal inhibitory concentration (MIC) of antimicrobial substances. *Nat Protoc* 3(2):163-175.
11. Nunn KL*, et al.* (2015) Enhanced Trapping of HIV-1 by Human Cervicovaginal Mucus Is Associated with Lactobacillus crispatus-Dominant Microbiota. *mBio* 6(5):e01084-01015.
12. Chan PP & Lowe TM (2009) GtRNAdb: a database of transfer RNA genes detected in genomic sequence. *Nucleic Acids Res* 37(Database issue):D93-97.
13. Yeoman CJ*, et al.* (2010) Comparative genomics of Gardnerella vaginalis strains reveals substantial differences in metabolic and virulence potential. *PLoS One* 5(8):e12411.
14. Bolger AM, Lohse M, & Usadel B (2014) Trimmomatic: a flexible trimmer for Illumina sequence data. *Bioinformatics* 30(15):2114-2120.
15. Kim D*, et al.* (2013) TopHat2: accurate alignment of transcriptomes in the presence of insertions, deletions and gene fusions. *Genome biology* 14(4):R36.
16. Anders S, Pyl PT, & Huber W (2015) HTSeq--a Python framework to work with high-throughput sequencing data. *Bioinformatics* 31(2):166-169.
17. Mentzel CM*, et al.* (2014) Wet-lab tested microRNA assays for qPCR studies with SYBR(R) Green and DNA primers in pig tissues. *Microrna* 3(3):174-188.
18. Cirera S & Busk PK (2014) Quantification of miRNAs by a simple and specific qPCR method. *Methods Mol Biol* 1182:73-81.
19. Livak KJ & Schmittgen TD (2001) Analysis of relative gene expression data using real-time quantitative PCR and the 2(-Delta Delta C(T)) Method. *Methods* 25(4):402-408.

20. Archer E (2013) rfPermute: Estimate permutation p-values for Random Forest importance metrics. in *R package version 1.5.2*.
21. Liaw A & Wiener M (2002) Classification and regression by randomForest. (R news), pp 18-22.
22. Grömping U (2009) Variable importance assessment in regression: linear regression versus random forest. *The American Statistician* 63(4):308-319.
23. Touw WG*, et al.* (2013) Data mining in the Life Sciences with Random Forest: a walk in the park or lost in the jungle? *Brief Bioinformatics* 14:315-326.
24. NCBI Resource Coordinators (2017) Database Resources of the National Center for Biotechnology Information. *Nucleic Acids Res* 45(D1):D12-D17.
25. Robinson MD, McCarthy DJ, & Smyth GK (2010) edgeR: a Bioconductor package for differential expression analysis of digital gene expression data. *Bioinformatics* 26(1):139-140.
26. Robinson MD & Oshlack A (2010) A scaling normalization method for differential expression analysis of RNA-seq data. *Genome biology* 11(3):R25.
27. Benjamini Y & Hochberg Y (1995) Controlling the False Discovery Rate: A Practical and Powerful Approach to Multiple Testing. *Journal of the Royal Statistical Society Series B (Methodological)* 57(1):289-300.
28. Goeman JJ & Solari A (2014) Multiple hypothesis testing in genomics. *Stat Med* 33(11):1946-1978.
29. Krämer A, Green J, Pollard J, & Tugendreich S (2014) Causal analysis approaches in Ingenuity Pathway Analysis. *Bioinformatics* 30(4):523-530.
30. Chou CH*, et al.* (2016) miRTarBase 2016: updates to the experimentally validated miRNA-target interactions database. *Nucleic Acids Res* 44(D1):D239-247.
31. Huang da W, Sherman BT, & Lempicki RA (2009) Systematic and integrative analysis of large gene lists using DAVID bioinformatics resources. *Nat Protoc* 4(1):44-57.
32. Schneider CA, Rasband WS, & Eliceiri KW (2012) NIH Image to ImageJ: 25 years of image analysis. *Nat Methods* 9(7):671-675.
33. Carpenter AE*, et al.* (2006) CellProfiler: image analysis software for identifying and quantifying cell phenotypes. *Genome biology* 7(10):R100.
