# Supplementary material for: The Cervicovaginal Microbiota-Host Interaction Modulates Chlamydia trachomatis Infection
Source: mBio. 2019 Aug 6;10(4):e01548-19. doi: 10.1128/mBio.01548-19 (PMC6692509; doi:10.1128/mBio.01548-19)
Supplement: FIG S3 [file mBio.01548-19-sf003.pdf]

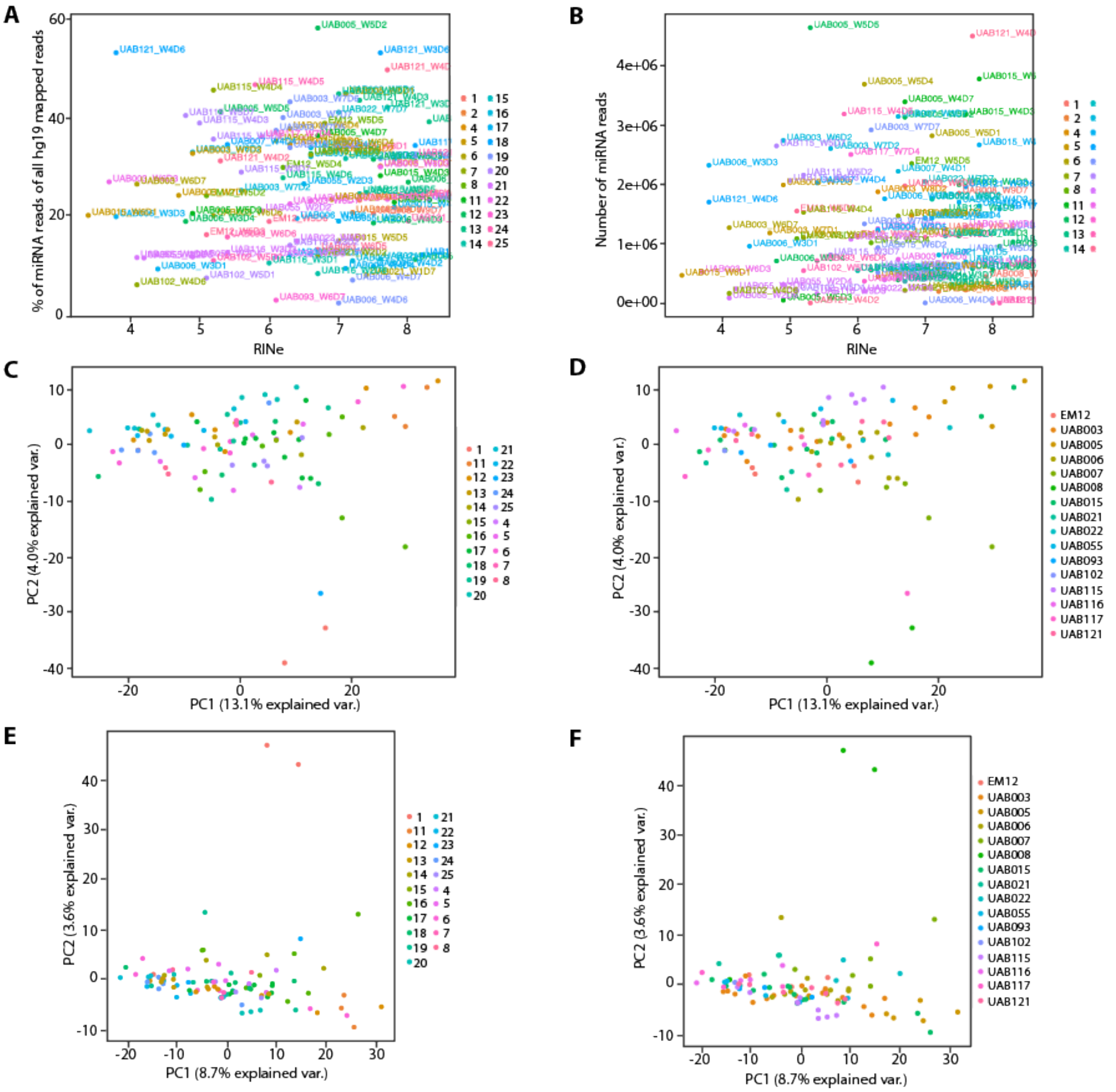


**Fig. S3.** Quality control (QC) of small RNA-seq samples. Relationship of total RNA quality (RINe, x-axis) versus miRNA mapped reads colored by sequencing batch as a percentage of all hg19 mapped reads (**A**) or total number of miRNA reads (**B**). PCA plot of log2 transformed miRNA read counts after low count read removal based on sequencing run (**C**) and subject (**D**). PCA plot of log2 transformed miRNA read counts after normalization colored by sequencing run (**E**) or subject (**F**). No statistically significant difference was observed based solely on any of the criteria tested.
