# Supplementary material for: The Cervicovaginal Microbiota-Host Interaction Modulates Chlamydia trachomatis Infection
Source: mBio. 2019 Aug 6;10(4):e01548-19. doi: 10.1128/mBio.01548-19 (PMC6692509; doi:10.1128/mBio.01548-19)
Supplement: FIG S4 [file mBio.01548-19-sf004.pdf]

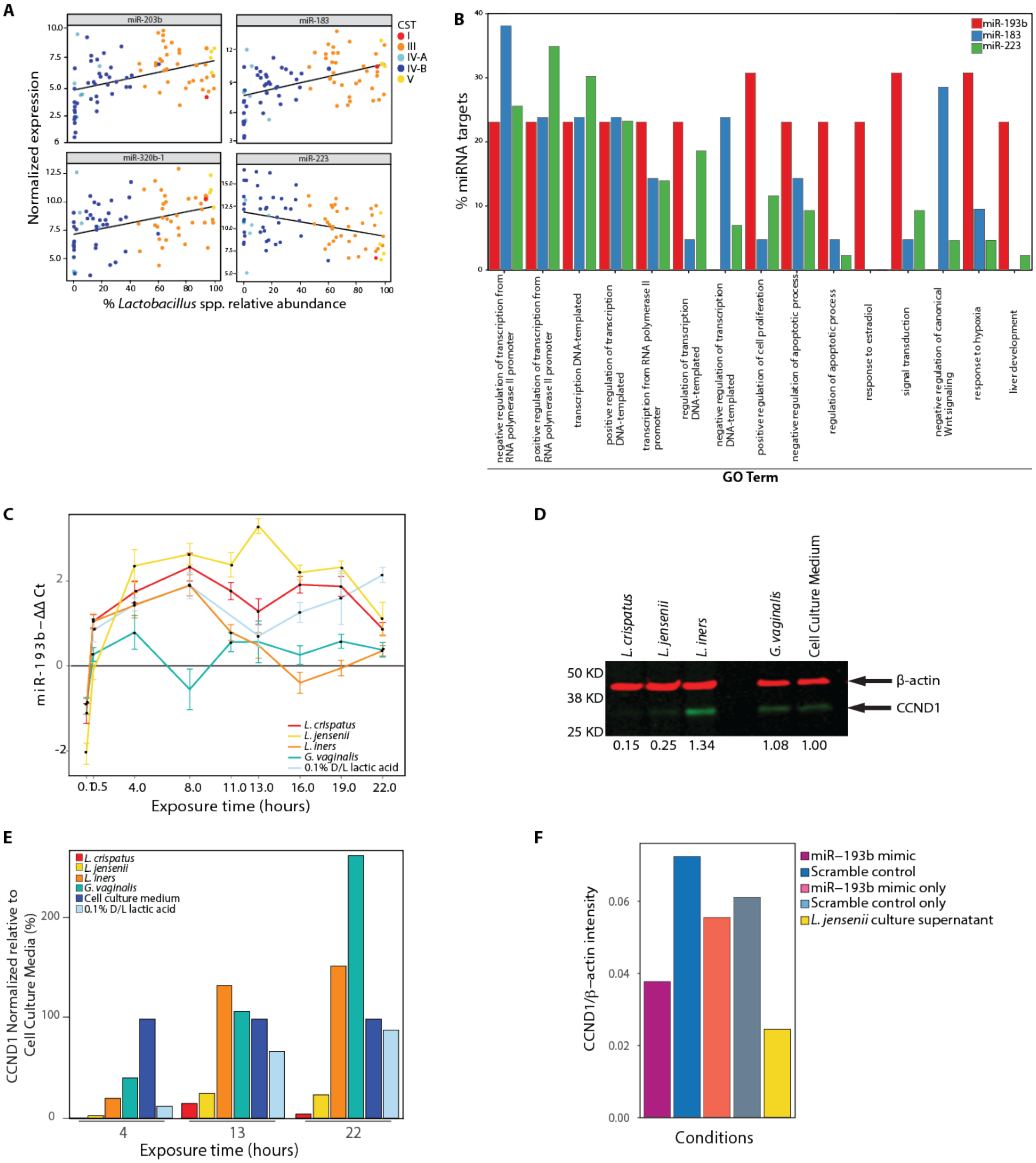

A
miR-203b
miR-183
miR-320b-1
miR-223
CST
I
III
IV-A
IV-B
V
Normalized expression
% Lactobacillus spp. relative abundance
B
miR-193b
miR-183
miR-223
% miRNA targets
negative regulation of transcription from RNA polymerase II promoter
positive regulation of transcription from RNA polymerase II promoter
transcription DNA-templated
positive regulation of transcription DNA-templated
transcription from RNA polymerase II promoter
regulation of transcription DNA-templated
negative regulation of transcription DNA-templated
positive regulation of cell proliferation
negative regulation of apoptotic process
regulation of apoptotic process
response to estradiol
signal transduction
negative regulation of canonical Wnt signaling
response to hypoxia
liver development
GO Term
C
miR-193b − ΔΔ Ct
L. crispatus
L. jensenii
L. iners
G. vaginalis
0.1% D/L lactic acid
Exposure time (hours)
D
L. crispatus
L. jensenii
L. iners
G. vaginalis
Cell Culture Medium
50 KD
38 KD
25 KD
β-actin
CCND1
0.15
0.25
1.34
1.08
1.00
E
L. crispatus
L. jensenii
L. iners
G. vaginalis
Cell culture medium
0.1% D/L lactic acid
CCND1 Normalized relative to Cell Culture Media (%)
Exposure time (hours)
F
miR−193b mimic
Scramble control
miR−193b mimic only
Scramble control only
L. jensenii culture supernatant
CCND1/β−actin intensity
Conditions

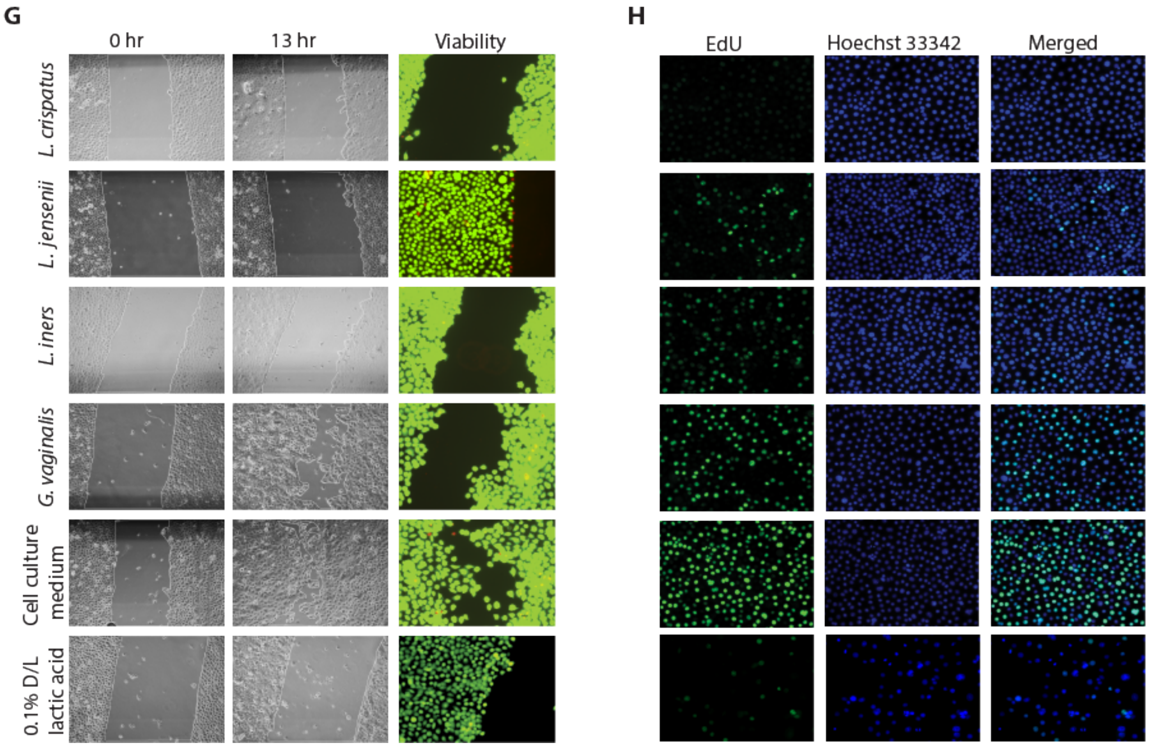

Fig. S4. A. and B. Association between *Lactobacillus* spp. relative abundance, miRs and predicted function. Small RNA sequencing analysis was performed on vaginal swab samples obtained from 16 women with varying CSTs and the correlation between vaginal *Lactobacillus* spp. relative abundance and the levels of miRs-203b, 183, 320b-1 and 223 expression was determined (**A**). Experimentally validated targets for miRs193b, 183, 223 were mapped to Gene Ontology (GO) DIRECT processes and the percentage of targets observed were mapped to the major TERMs (**B**). miRs 203b and 320b-1 did not have any experimentally validated targets as per the current literature. **C. D. E. and F.** miR-193b expression and CCND1 levels in VK2 epithelial cells. Expression levels of miR-193b after exposure to various culture supernatants were compared to cell culture medium exposed sample reference across 9 time points spanning 22h. Time points at which statistical significance was obtained are shown as black points (p-value≤0.05) (**C**). Representative western blot of CCND1 (36 KD) after 13h culture supernatant exposure in VK2 cells (**D**). Protein expression levels of CCND1 (36 KD) after 4h, 13h and 22h culture supernatant exposure in VK2 cells normalized to β-actin level with cells exposed only to culture medium used as reference (**E**). CCND1 protein levels of VK2 cells exposed to various transfection controls and normalized to β-actin (**F**). **G. and H.** VK2 cell migration and proliferation assay. VK2 epithelial cells were scratched and exposed to respective culture supernatants for 13 hours (13h). Representative images from scratch assay microscopy at 100X of VK2 cells exposed to 20% culture supernatant of *L. crispatus, L. jensenii, L. iners, G. vaginalis*, cell culture medium or 0.1% D/L lactic acid at 0 h and 13 h post exposure (A). Representative images of cell viability 13 h post exposure (green is viable, red is non-viable) are also shown (**G**). Proliferation of VK2 cells after 13 h exposure to bacterial culture supernatants was determined by staining for new DNA synthesis (EdU nucleobases positive staining - green), while total cell number was determined by nucleus number (Hoechst staining - blue), and merged images of both were created (**H**).
