# Supplementary material for: The Cervicovaginal Microbiota-Host Interaction Modulates Chlamydia trachomatis Infection
Source: mBio. 2019 Aug 6;10(4):e01548-19. doi: 10.1128/mBio.01548-19 (PMC6692509; doi:10.1128/mBio.01548-19)
Supplement: FIG S5 [file mBio.01548-19-sf005.pdf]

UNIVERSITY of MARYLAND
SCHOOL OF MEDICINE

RESEARCH USE ONLY

Biopolymer-Genomics Core Laboratory
655 West Baltimore St.
Bressler Research Bldg. 7-036
Baltimore, MD 21201
410 706 8553
biopolymer@som.umaryland.edu
http://medschool.umaryland.edu/Biopolymer

CELL LINE AUTHENTICATION REPORT

Allele Table

Locus	BJ	CELLOSAURUS Reference BJ [Human fibroblast] (RRID:CVCL_3653)
Amelogenin	X	X
CSF1PO	10,12	10,12
D13S317	8,9	8,9
D16S539	9,13	9,13
D21S11	28,29	NO DATA
D5S818	12	12
D7S820	11,12	11,12
TH01	7,8	7,8
TPOX	10,11	10,11
vWA	16,18	16,18

☐The submitted sample profile is human, but not a match for any profile in the CELLOSAURUS STR database.

☒The submitted profile is an exact match for the following CELLOSAURUS human cell line(s) in the CELLOSAURUS STR database (8 core loci plus Amelogenin): **BJ [Human fibroblast] (RRID:CVCL_3653)**

☐The submitted profile is similar to the following CELLOSAURUS human cell line(s).

☐The submitted cell line is contaminated, and has tested positive for mouse marker.

Interpretation
The samples provided exhibit identical genetic profiles.
Sample BJ shares 15 alleles of 15 alleles (100.0%) with the CELLOSAURUS Reference.

Allele Table

Locus	VK2	ATCC Reference CRL-2616(VK2)
Amelogenin	X	X
CSF1PO	10,11	10,11
D13S317	9,12	9,12
D16S539	9	9
D21S11	29,31.2	NO DATA
D5S818	9,10	9,10
D7S820	10,11	10,11
TH01	7,9.3	7,9.3
TPOX	11	11
vWA	16	16

☐The submitted sample profile is human, but not a match for any profile in the ATCC STR database.

☒The submitted profile is an exact match for the following ATCC human cell line(s) in the ATCC STR database (8 core loci plus Amelogenin): **CRLR 2616(VK2)**

☐The submitted profile is similar to the following ATCC human cell line(s).

☐The submitted cell line is contaminated, and has tested positive for mouse marker.

Interpretation
The sample provided exhibits identical genetic profiles.
Sample MRC5 shares 13 alleles of 13 alleles (100.0 %) with the ATCC reference.

Explanation of Test Results
Cell lines with ≥80% match are considered to be related; i.e., derived from a common ancestry. A cell line with an STR profile match of ≤56% is considered unrelated. A unique cell line has a STR profile that is different from another unique cell line.

STR typing was performed using the Promega Geneprint 10 System™. The kit includes 10 human specific loci for human cell line authentication. The human loci collectively provide a genetic profile with a random match probability of 1 in 2.92×10^9. Where more than one human profile is observed, alleles of the minor contributor are indicated in parentheses.
. Our laboratory uses GenePrint® 5X Mouse Primer Pair Mix is designed to be used as a sensitive marker that specifically detects the presence of mouse (*Mus musculus*) DNA while simultaneously providing detection of about 1% fraction of mouse contaminant in a human cell line when using extracted DNA.

UNIVERSITY of MARYLAND
SCHOOL OF MEDICINE

Biopolymer-Genomics Core Laboratory
655 West Baltimore St.
Bressler Research Bldg. 7-036
Baltimore, MD 21201
410 706 8553
biopolymer@som.umaryland.edu
http://medschool.umaryland.edu/Biopolymer

Report Date: 11/07/2016

Sample (s) received Date: 11/04/2016

Name of Requester: Steven Smith

Cell Line ID: A2EN P62

Biopolymer Lab Order: GCF-SS-1050

CELL LINE AUTHENTICATION REPORT

RESEARCH USE ONLY

Allele Table

Locus	A2EN P62
Amelogenin	X
CSF1PO	12,13
D13S317	9,13
D16S539	9,11
D21S11	29,32.2
D5S818	11,13
D7S820	11
TH01	6
TPOX	8,11
vWA	17

Interpretation
Sample SS-A2EN P62 did not match with any ATCC Reference.

Explanation of Test Results
Cell lines with ≥80% match are considered to be related; i.e., derived from a common ancestry. A cell line with an STR profile match of ≤56% is considered unrelated. A unique cell line has a STR profile that is different from another unique cell line.

- ☐ The submitted sample profile is human, but not a match for any profile in the ATCC STR database.
- ☐ The submitted profile is an exact match for the following ATCC human cell line(s) in the ATCC STR database (8 core loci plus Amelogenin):
- ☐ The submitted profile is similar to the following ATCC human cell line(s).

- ☐ The submitted cell line is contaminated, and has tested positive for mouse marker.

STR typing was performed using the Promega Geneprint 10 System™. The kit includes 10 human specific loci for human cell line authentication. The human loci collectively provide a genetic profile with a random match probability of 1 in 2.92 × 10^9. Where more than one human profile is observed, alleles of the minor contributor are indicated in parentheses.
. Our laboratory uses GenePrint® 5X Mouse Primer Pair Mix is designed to be used as a sensitive marker that specifically detects the presence of mouse (*Mus musculus*) DNA while simultaneously providing detection of about 1% fraction of mouse contaminant in a human cell line when using extracted DNA.

Fig. S5. Validation certificate for A2EN, VK2 and BJ cell lines.
